# Ruthenium (II) complex *cis*-[Ru^II^(ŋ^2^-O_2_CC_7_H_7_O_2_)(dppm)_2_]PF_6_-hmxbato induces ROS-mediated apoptosis in lung tumor cells producing selective cytotoxicity

**DOI:** 10.1038/s41598-020-72420-w

**Published:** 2020-09-21

**Authors:** Mônica Soares Costa, Yasmim Garcia Gonçalves, Bruna Cristina Borges, Marcelo José Barbosa Silva, Martin Krähenbühl Amstalden, Tássia Rafaella Costa, Lusânia Maria Greggi Antunes, Renata Santos Rodrigues, Veridiana de Melo Rodrigues, Eduardo de Faria Franca, Mariana Alves Pereira Zoia, Thaise Gonçalves de Araújo, Luiz Ricardo Goulart, Gustavo Von Poelhsitz, Kelly Aparecida Geraldo Yoneyama

**Affiliations:** 1 Laboratório de Bioquímica e Toxinas Animais, Instituto de Biotecnologia, Universidade Federal de Uberlândia, UFU, Pará avenue, 1720, Uberlândia, MG CEP 38400-902 Brazil; 2 Instituto de Química, Universidade Federal de Uberlândia, UFU, Uberlândia, MG Brazil; 3 Laboratório de Osteoimunologia e Imunologia dos Tumores, Instituto de Ciências Biomédicas, Universidade Federal de Uberlândia, UFU, Uberlândia, MG Brazil; 4 Departamento de Análises Clínicas, Toxicologia e Ciências Alimentares, Faculdade de Ciências Farmacêuticas de Ribeirão Preto, Universidade de São Paulo, Ribeirão Preto, São Paulo CEP 14040-903 Brazil; 5 Laboratório de Cristalografia e Química Computacional, Instituto de Química, Universidade Federal de Uberlândia, UFU, Uberlândia, MG Brazil; 6 Laboratório de Nanobiotecnologia, Instituto de Biotecnologia, Universidade Federal de Uberlândia, UFU, Uberlândia, MG Brazil

**Keywords:** Biochemistry, Cancer, Cell biology

## Abstract

Ruthenium complexes have been extensively explored as potential molecules for cancer treatment. Considering our previous findings on the remarkable cytotoxic activity exhibited by the ruthenium (II) complex 3-hydroxy-4-methoxybenzoate (hmxbato)-*cis*-[Ru^II^(ŋ^2^-O_2_CC_7_H_7_O_2_)(dppm)_2_]PF_6_ against *Leishmania* promastigotes and also the similar metabolic characteristics between trypanosomatids and tumor cells, the present study aimed to analyze the anticancer potential of hmxbato against lung tumor cells, as well as the partial death mechanisms involved. Hmxbato demonstrated selective cytotoxicity against A549 lung tumor cells. In addition, this complex at a concentration of 3.8 µM was able to expressively increase the generation of reactive oxygen species (ROS) in tumor cells, causing an oxidative stress that may culminate in: (1) reduction in cellular proliferation; (2) changes in cell morphology and organization patterns of the actin cytoskeleton; (3) cell arrest in the G2/M phase of the cell cycle; (4) apoptosis; (5) changes in the mitochondrial membrane potential and (6) initial DNA damage. Furthermore, we demonstrated that the induction of programmed cell death can occur by the intrinsic apoptotic pathway through the activation of caspases. It is also worth highlighting that hmxbato exhibited predominant actions on A549 tumor cells in comparison to BEAS-2B normal bronchial epithelium cells, which makes this complex an interesting candidate for the design of new drugs against lung cancer.

## Introduction

Cancer is one of the most common causes of death worldwide and despite advances in the development of new therapies, it is still a major concern for health systems. Among cancer types, lung cancer is the most frequently diagnosed, accounting for 11.6% of all reported cancer cases worldwide. In addition, lung cancer is the third leading cause of death by cancer^1,2^.

In recent years, pharmacological treatments of various types of cancer have been conducted with platinum-based drugs such as cisplatin, carboplatin, and oxaliplatin. However, numerous disadvantages are associated with the use of cisplatin and its derivatives as antitumor agents, namely: (1) drug resistance, which can be developed after treatment; (2) low toxicity against some types of cancer, such as cervical, lung and pancreatic cancer; and (3) low selectivity for tumor cells, thus triggering several side effects. These disadvantages have encouraged the development of new metal complex-based chemotherapeutic agents that have higher and more selective toxicities on tumor cells^3,4^.

In this sense, ruthenium complexes have gained importance in recent years as promising candidates for anticancer therapy due to their numerous advantages. One of the main properties related to the ruthenium complex is its octahedral geometry that provides a vast repertoire for the formation of new complexes and may increase its degree of selectivity. In addition, the different ruthenium oxidation stages (II and III) and the ease of modulation between metal–ligand facilitate the interaction of complexes with target molecules. Finally, ruthenium is also interesting due to its low toxicity, good stability and ability to mimic iron by binding to plasma proteins such as albumin and ferritin^3,5,6^.

The development of new antitumor drugs requires understanding the mechanisms of action of new pharmacological candidates, which highlights the importance of elucidating the molecular and biochemical mechanisms involved. Recent studies have shown that some ruthenium complexes target the genomic DNA and may cause cell cycle arrest, ROS-mediated mitochondrial alterations and, finally, cell death by apoptosis^3,7–9^.

Previous studies by our research group showed a remarkable cytotoxic activity for the ruthenium complex called 3-hydroxy-4-methoxybenzoate (hmxbato)-*cis*-[Ru^II^(ŋ^2^-O_2_CC_7_H_7_O_2_)(dppm)_2_]PF_6_ against promastigote forms of different *Leishmania* species^10^. In addition, we have described that the hmxbato complex was able to induce increased ROS levels and cause death of *Leishmania (Leishmania) amazonensis* promastigotes by apoptosis^11^*.* Other authors have also demonstrated the anti-*Leishmania* potential of ruthenium complexes^12–14^.

Given the remarkable cytotoxic activity exhibited by hmxbato against *Leishmania* promastigotes, associated with the similarity of some metabolic characteristics between trypanosomatids and tumor cells, such as high replicability and high energy and nutrient requirements^15,16^, the present study had as objective the evaluation of the anticancer potential of hmxbato against lung tumor cells, as well as the death mechanisms involved. Simultaneously, all assays were also performed with normal lung cells to verify the potential of this molecule as a candidate for prospecting novel antitumor drugs.

## Results

### Hmxbato exhibits high selectivity and cytotoxic potential against A549 tumor cells

**Figure 1:**
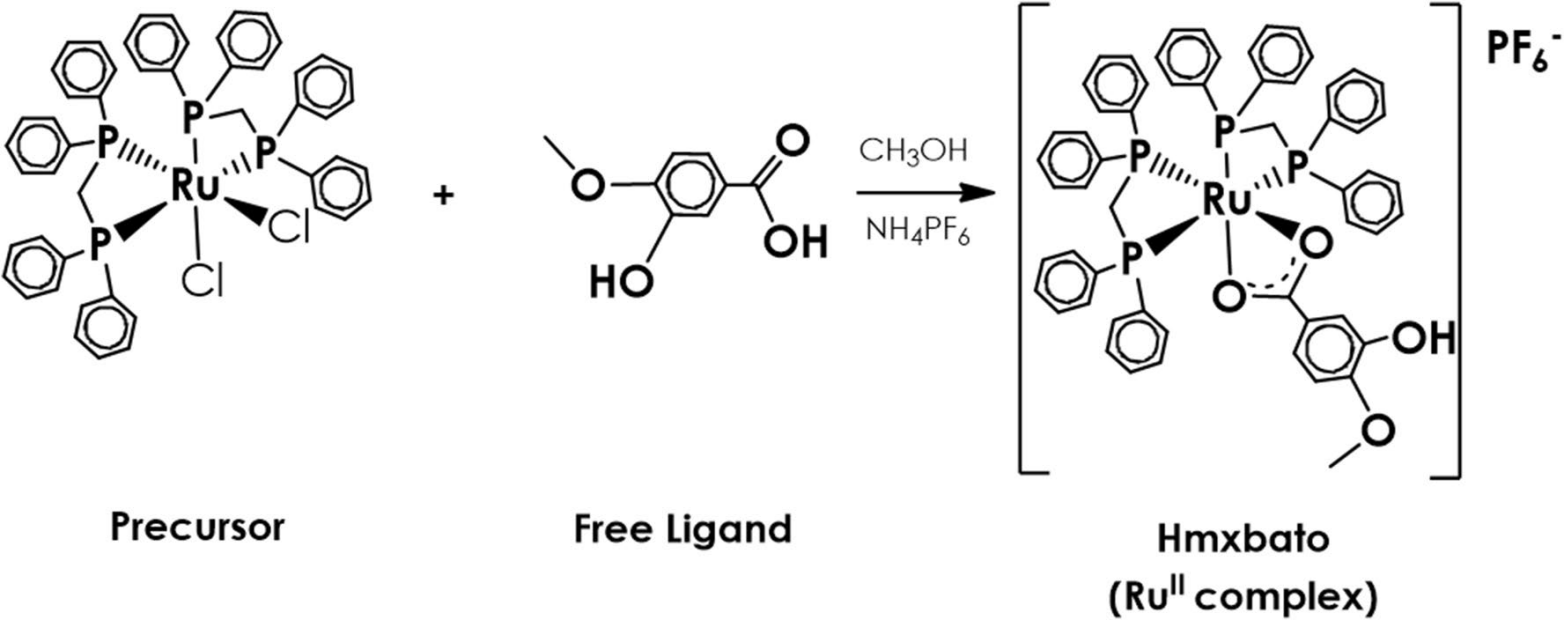
Synthetic route of the ruthenium complex *cis*-[Ru^II^(ŋ^2^-O_2_CC_7_H_7_O_2_)(dppm)_2_]PF_6_—hmxbato, where dppm = bis(diphenylphosphino)methane.

The in vitro cytotoxicity of hmxbato complex, its metal precursor and free ligand (Fig. 1) was determined by MTT assay. Additionally, the cisplatin cytotoxicity was also evaluated for comparison purposes. IC_50_ values for tumor (A549) and normal (BEAS-2B) lung cells were expressed in Table 1. Hmxbato and its precursor showed considerable cytotoxic activity against A549 tumor cells, exhibiting IC_50_ values of 3.8 µM and 21.2 µM, respectively. Hmxbato presented higher cytotoxicity than cisplatin drug, which displayed an IC_50_ value of 26.4 µM. As expected, the free ligand showed no cytotoxic activity. Thus, comparatively, hmxbato exhibited higher cytotoxic potential when compared to cisplatin (~ 6 times lower IC_50_ value) or its precursor (~ 5 times lower IC_50_ value). Moreover, the cytotoxicity of compounds was also evaluated in normal lung cells (BEAS-2B). Hmxbato, precursor and cisplatin exhibited IC_50_ values of 40.3 µM, 29.2 µM and 13.3 µM, respectively. The selectivity cytotoxicity index (ICS), calculated as the ratio of the IC_50_ values for normal and tumor cells, showed values of 0.5 for cisplatin, 1.4 for the precursor and 10.6 for hmxbato. Taken together, the results show that the metal–ligand association resulted in an effective complex against A549 lung tumor cells.

**Table 1:** Cytotoxic effects of hmxbato on tumor and normal lung cells and their respective IC_50_ and SCI values.

	IC_50_ (95% CI)^a^, µM*	IC_50_ (95% CI), µM*	Selective Cytotoxicity index (SCI)^b^
Treatment	A549	BEAS	
Cisplatin	26.4 (21.1–33.0)	13.38 (10.1–17.7)	0.5
Precursor	21.2 (16.0–28.1)	29.2 (22.3–38.2)	1.4
Free ligand	˂ 200	˂ 200	Not determined
Hmxbato	3.8 (2.3–6.2)	40.3 (22.6–71.7)	10.6

^a^Inhibitory concentration for 50% of cells.

^b^SCI = IC_50_ of non-cancerous BEAS-2B-cells/IC_50_ of cancer A549 cells.

*IC_50_ values were calculated from concentrations: 200; 100; 50; 25; 12.5; 6.25; 3.12; 1.56; 0.78; 0.39; 0.19 and 0.097 μM.

### Hmxbato interferes with A549 tumor cell proliferation and recovery

**Figure 2:**
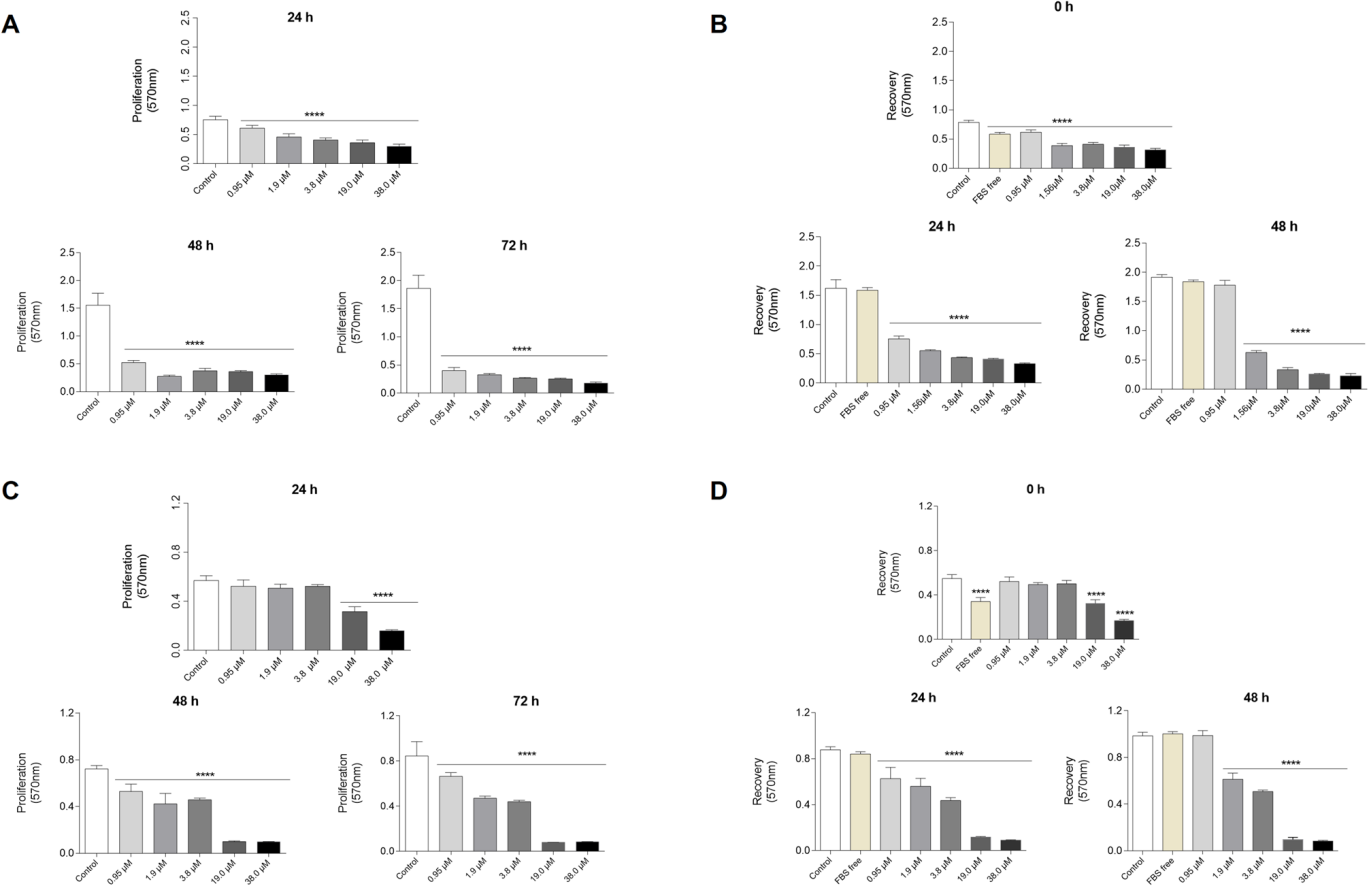
Effects of hmxbato on the proliferation and recovery of tumor and normal lung cells. Tumor (A549) and normal (BEAS-2B) lung cells were incubated for 24 h in the absence (control) or presence of different concentrations of hmxbato (0.95 µM, 1.9 µM, 3.8 µM, 19 µM and 38 µM). The proliferation of A549 (**A**) and BEAS-2B (**C**) cells was evaluated after 24, 48 and 72 h of treatment. Recovery from the cell proliferation induced by the hmxbato treatment was also assessed. For that, A549 and BEAS-2B cells treated with complete medium (control), FBS free medium and different concentrations of hmxbato (0.95 µM, 1.9 µM, 3.8 µM, 19 µM and 38 µM) for 24 h were washed with PBS and incubated with complete medium. The recovery of A549 (**B**) and BEAS-2B (**D**) cells was evaluated at 0, 24 and 48 h. Data were expressed as mean ± standard deviation of experiments performed in triplicate. Three independent experiments were performed. Statistical differences were determined using one-way ANOVA and Tukey's multiple comparison test. Significant differences with respect to control were considered when *p* < 0.1 (*), *p* < 0.01 (**), *p* < 0.001 (***) and *p* < 0.0001 (****).

After determining the hmxbato IC_50_ value for A549 tumor cells, five different concentrations corresponding to the ¼ IC_50_ (0.95 µM), ½ IC_50_ (1.9 µM), IC_50_ (3.8 µM), 5 × IC_50_ (19 µM) and 10 × IC_50_ (38 µM) values were used to evaluate the complex effects in proliferation and recovery of tumor and normal lung cells. Hmxbato significantly inhibited A549 cell proliferation at all concentrations and times assessed (Fig. 2A). The removal of hmxbato from the culture medium was not able to recover the proliferation of A549 cells, except for cells grown in presence of hmxbato at 0.95 µM after 48 h recovery (Fig. 2B). The proliferation assay of BEAS-2B showed that hmxbato at 0.95 µM, 1.9 µM and 3.8 µM promotes no antiproliferative effect after 24 h treatment (Fig. 2C). However, higher concentrations (19 µM and 38 µM) caused statistically significant inhibitions in proliferation at all times tested (24, 48 and 72 h). The recovery proliferation assay of BEAS-2B showed that hmxbato promoted statistically significant interference in the recovery cells (Fig. 2D), except for cells cultured in presence of hmxbato in the lowest concentration (0.95 µM) after 48 h recovery. Therefore, hmxbato significantly interfered with both proliferation and recovery of lung tumor cells. However, the complex did not interfere with normal lung cell proliferation at concentrations of 0.95 µM, 1.9 µM and 3.8 µM (IC_50_ value for tumor cell A549) after 24 h of treatment.

### Hmxbato prevents the clonogenic ability of A549 tumor cells

**Figure 3:**
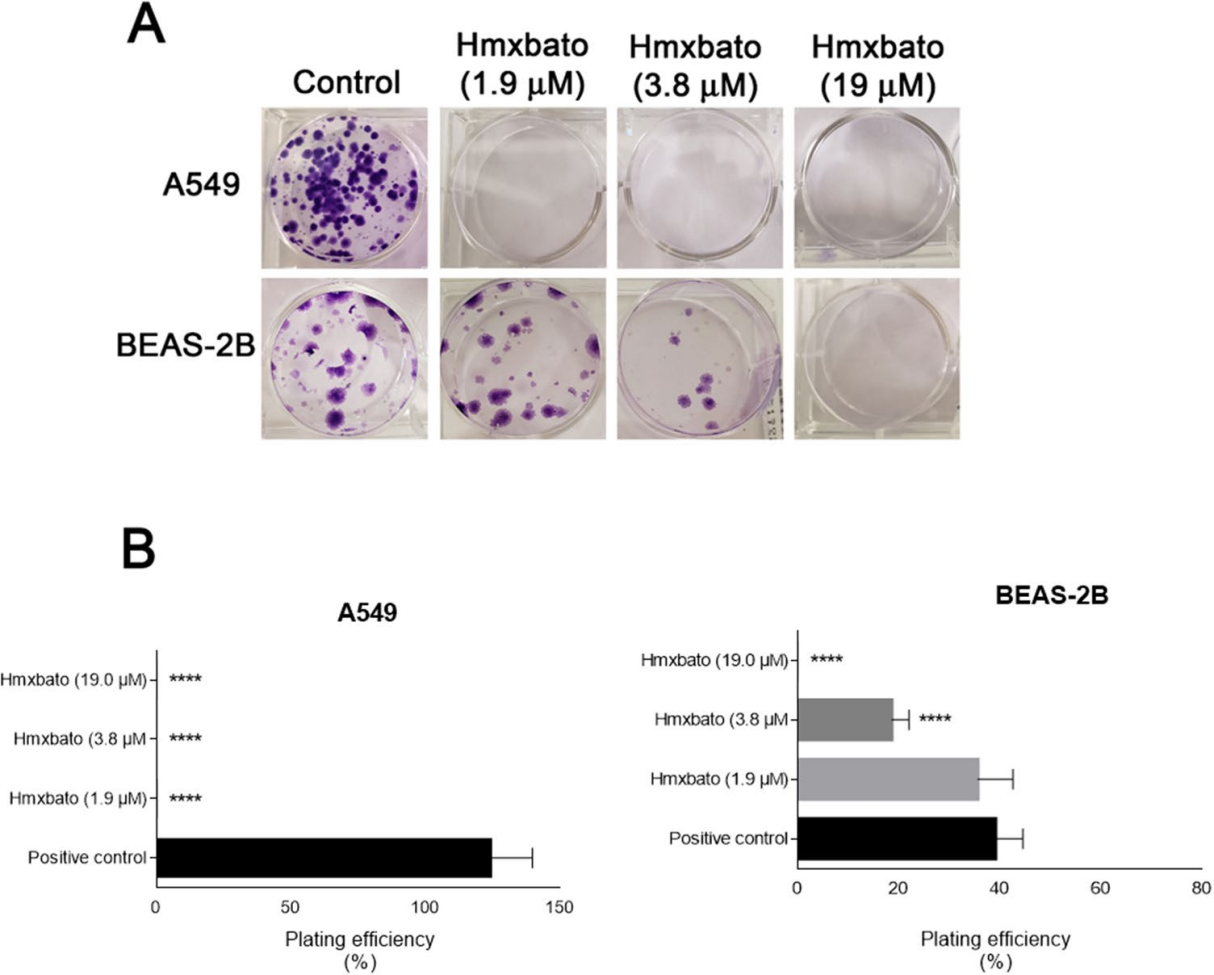
Hmxbato prevented the clonogenic growth of lung tumor cells. Tumor (A549) and normal (BEAS-2B) lung cells were incubated for 24 h in the absence (control) or presence of hmxbato (1.9 µM, 3.8 µM and 19 µM). (**A**) Representative images of an experiment developed in 6-well plates showing the formation of A549 and BEAS-2B cell colonies after removal of treatments and incubation with culture medium for 14 days. (**B**) Bar graphs showing plating efficiency (%), calculated by the number of colonies formed / number of cells seeded × 100%. Data were expressed as mean ± standard deviation of experiments performed in triplicate. Three independent experiments were performed. Statistical differences were determined using one-way ANOVA and Tukey's multiple comparison test. Significant differences with respect to control were considered when *p* < 0.1 (*), *p* < 0.01 (**), *p* < 0.001 (***) and *p* < 0.0001 (****).

Considering the hmxbato effects on recovery of cell proliferation and aiming to analyze the complex effects on colony-forming capacity of cells, a clonogenic assay was performed. Thus, A549 and BEAS-2B cells incubated in the absence (control) and presence of hmxbato at 0.5 × IC_50_ (1.9 µM), 1 × IC_50_ (3.8 µM) and 5 × IC_50_ (19 µM) concentrations for 24 h were washed, incubated with complete culture medium and analyzed for colony-forming capacity after 14 days (Fig. 3). A549 cells were unable to form colonies after treatment with the complex. In contrast, BEAS-2B cells exhibited colony formation after treatment with the complex at concentrations of 1.9 µM and 3.8 µM (Fig. 3A). Furthermore, for BEAS-2B cells, no statistically significant difference was observed between the lowest concentration of hmxbato (1.9 µM) and control cells (Fig. 3B). These results demonstrate that the complex, even at low concentrations, was able to prevent the clonogenic capacity of A549 tumor cells, while for BEAS-2B non-tumor cells the colony-forming capacity was only slightly affected.

### Hmxbato induces tumor cell arrest in the G2/M phase of the cell cycle

**Figure 4:**
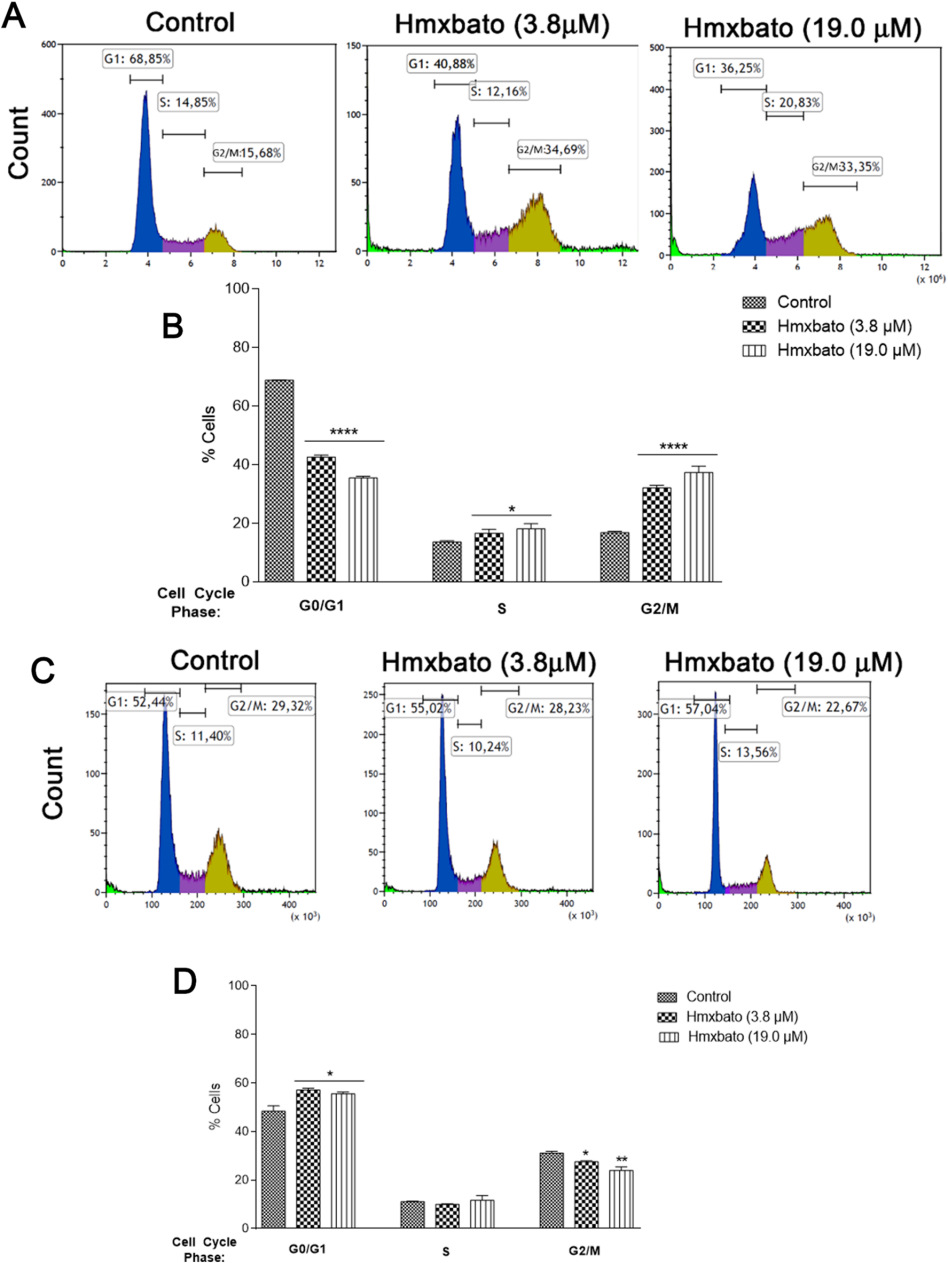
Hmxbato caused cell cycle arrest in the G2/M phase. Tumor (A549) and normal (BEAS-2B) lung cells were incubated for 24 h in the absence (control) or presence of hmxbato (3.8 µM and 19 µM) and subjected to cell cycle analysis. (**A**) Histograms of one of the independent experiments performed, showing the distribution of cells in the phases of the cell cycle. Distribution of A549 and BEAS-2B cells according to DNA content after PI staining. Fluorescence was measured by flow cytometry. (**B**) Bar graphs showing the percentages of A549 and BEAS-2B cells in each subpopulation of the cell cycle phases: G0/G1, S, G2/M. Data were expressed as mean ± standard deviation of experiments performed in triplicate. Three independent experiments were performed and over 10,000 cells were analyzed in each replicate. Statistical differences were determined using one-way ANOVA and Tukey's multiple comparison test. Significant differences with respect to control were considered when *p* < 0.1 (*), *p* < 0.01 (**), *p* < 0.001 (***) and *p* < 0.0001 (****).

After exposure to hmxbato at different concentrations, A549 tumor cells showed an irregular distribution of cells at different stages of the cell cycle compared to the distribution of untreated tumor cells (control) (Fig. 4A). Results showed a G1 phase cell reduction from 68.85% (control cells) to 40.88% and 36.25% after treatment with the complex at concentrations of 3.8 µM and 19 µM, respectively (Fig. 4B). Quantitative analysis, based on three independent experiments, showed that treatment with hmxbato at both concentrations increased the percentage of A549 tumor cells in the G2/M phase, with 16.75% of control cells in this phase in contrast to 32.00% and 37.24% when cells were treated with 3.8 µM and 19 µM of the complex, respectively. Also, no statistically significant differences were observed between the concentrations of hmxbato analyzed. In BEAS-2B non-tumor cells, hmxbato only promoted slight changes in cell distribution at different stages of the cell cycle (Fig. 4C). In this case, the quantitative analysis (Fig. 4D) of three independent experiments showed small decreases in the percentage of cells in the G2/M phase in relation to the control, decreasing from 31% (control) to 29.4% (3.8 µM) and 23.9% (19 µM). Thus, the results show that hmxbato was capable of inducing an expressive arrest of A549 cells in the G2/M phase of the cell cycle.

### Hmxbato induces apoptosis in A549 tumor cells

**Figure 5:**
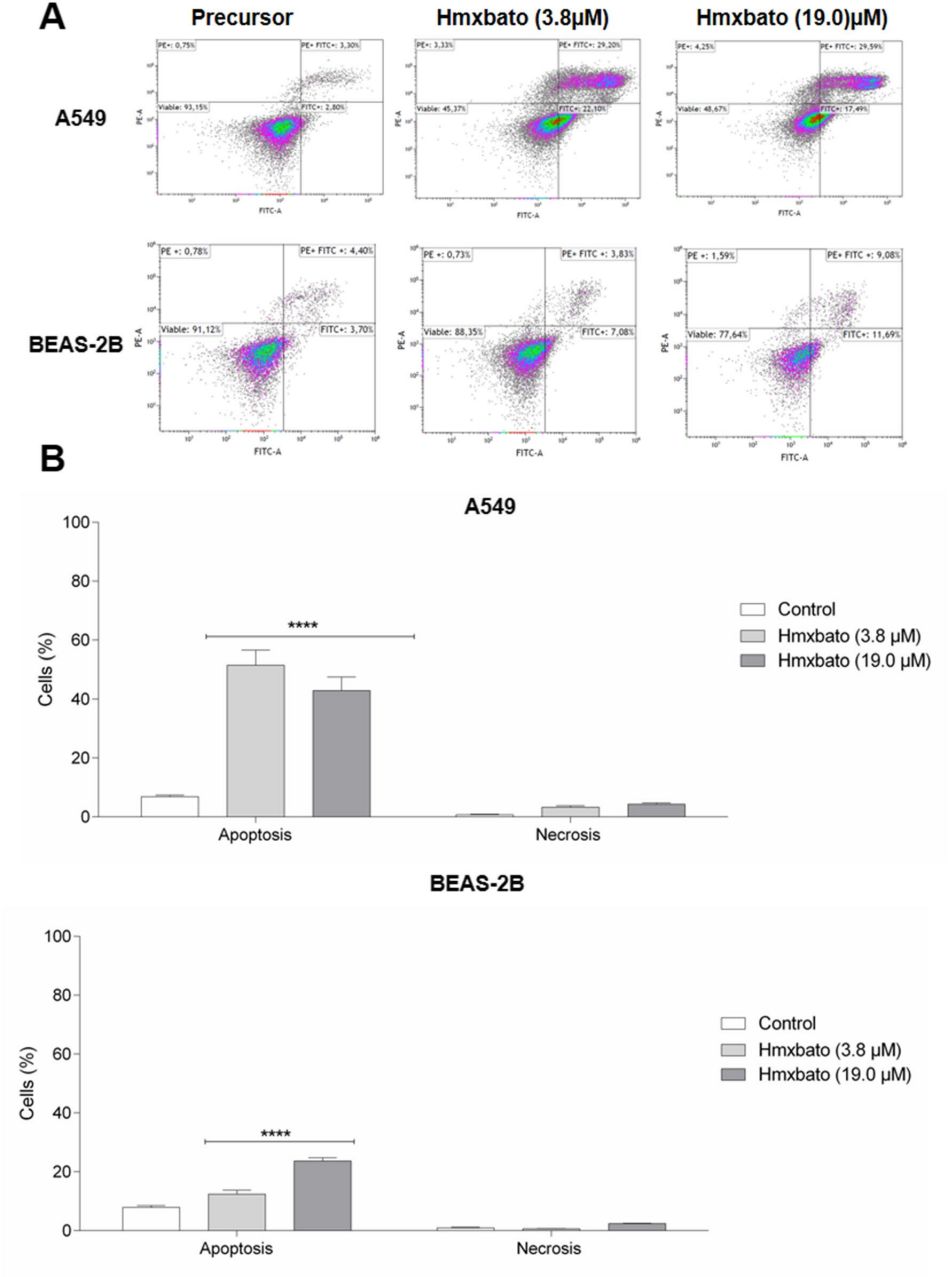
Hmxbato induced apoptosis in lung tumor cells. Tumor (A549) and normal (BEAS-2B) lung cells were incubated for 24 h in the absence (control) or presence of hmxbato (3.8 µM and 19 µM) and subjected to annexin V-FITC and propidium iodide double-staining assay. (**A**) Dot plot graphs, referring to one of the independent experiments performed, showing the distribution of A549 and BEAS-2B cells in different quadrants, according to the incorporation or not of Annexin V (AV) FITC/propidium iodide (PI). Lower left quadrant, viable cells; right lower quadrant [AV(+)], initial apoptosis; right upper quadrant [AV/PI(+)], late apoptosis; upper left quadrant [PI(+)], necrosis. (**B**) Bar graphs showing the quantification of A549 and BEAS-2B cells in apoptosis/necrosis. Data were expressed as mean ± standard deviation of experiments performed in triplicate. Three independent experiments were performed and over 10,000 cells were analyzed in each replicate. Statistical differences were determined using one-way ANOVA and Tukey's multiple comparison test. Significant differences with respect to control were considered when *p* < 0.1 (*), *p* < 0.01 (**), *p* < 0.001 (***) and *p* < 0.0001 (****).

Both the 3.8 µM and 19 µM concentrations of ruthenium (II) complex hmxbato were examined for their capacity to induce phosphatidylserine externalization on A549 cells, which is a hallmark of apoptosis. For this purpose, the annexin V-FITC/PI assay and flow cytometer was used, as previously reported^17^. The two-parameter dot plot histogram analysis (Fig. 5A) showed a clear shift in the distribution profiles of the cells, mainly located towards the lower and upper right quadrants, which are related to cells in early and late apoptosis, respectively. In contrast, hmxbato-treated BEAS-2B cells did not exhibit major changes in their distribution profile, with most remaining in the quadrant for viable cells. The values shown in the dot plots, obtained in independent experiments, were analyzed and used for the quantification of cells in apoptosis/necrosis and are shown in Fig. 5B. Treatment of A549 cells with hmxbato at concentrations of 3.8 µM and 19 µM induced apoptosis (early and late) in 51.46% and 42.78% of cells, respectively. On the other hand, hmxbato induced apoptosis (early and late) in only 12.3% and 23.6% of BEAS-2B cells after treatment with 3.8 µM and 19 µM, respectively. These results indicate that hmxbato, at the concentration of 3.8 µM, was capable of inducing apoptosis in a significant percentage of tumor cells, while this same concentration of the complex only induced apoptosis in a small percentage of non-tumor cells.

### Hmxbato promotes changes in A549 tumor cell morphology associated with cytoskeleton alterations

**Figure 6:**
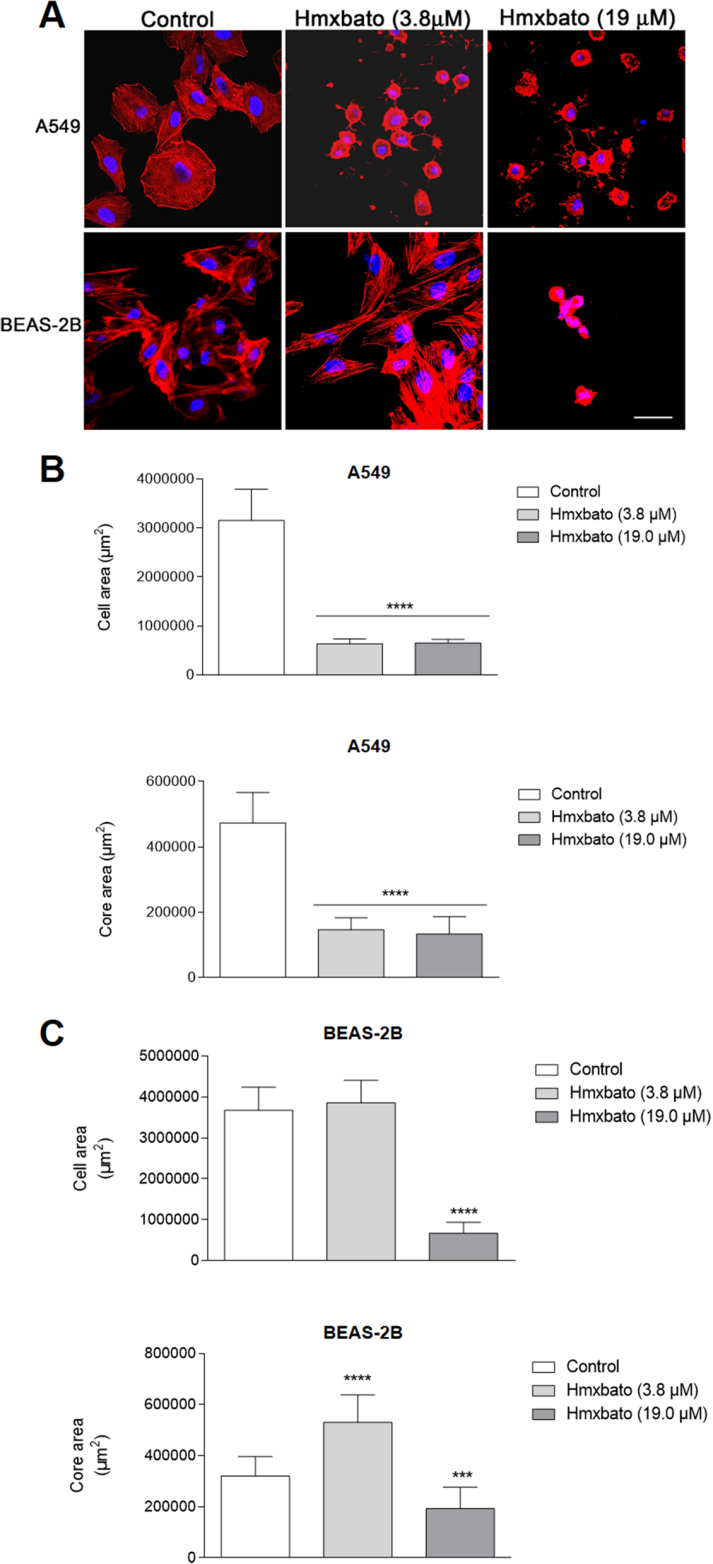
Hmxbato induced changes in lung tumor cell morphology and cytoskeleton. Tumor (A549) and normal (BEAS-2B) lung cells were cultured for 24 h in the absence (control) or presence of hmxbato (3.8 µM and 19 µM) and subjected to confocal microscopy analysis. (**A**) The panels show cells labeled with phalloidin-Atto 565 (red) and To-Pro (blue). The images are representative of three independent experiments. Bar: 20 µm. Additionally, the area (size) and the nucleus (**B**) A549 and (**C**) BEAS-2B cells were manually measured using the ImageJ software (National Institutes of Health, USA). The results were transferred to the GraphPad Prism software version 6.01 software, where the graphs were built and statistical analyses were performed. Data were expressed as mean ± standard deviation of experiments carried out in triplicate. Three independent experiments were performed and more than 10,000 cells were analyzed in each triplicate. Statistical differences were determined using one-way ANOVA and Tukey's multiple comparison test. Significant differences with respect to control were considered when *p* < 0.1 (*), *p* < 0.01 (**), *p* < 0.001 (***) and *p* < 0.0001 (****).

To assess whether hmxbato promotes changes in cell morphology and cytoskeleton, A549 and BEAS-2B cells were stained with phalloidin after treatment with the complex at different concentrations (Fig. 6). The images showed that hmxbato promoted significant changes in the morphology and cytoskeleton of A549 tumor cells (Fig. 6A), with the most evident alterations being: (1) reduction of cell density; (2) increase in the condensation of actin filaments, and (3) cell rounding. In addition, there was a statistically significant reduction (Fig. 6B) in cell size (represented by cell area) and nucleus size at the two tested concentrations. In contrast, the treatment of BEAS-2B cells with the complex at a concentration of 3.8 µM did not result in major changes in the cytoskeleton and cell morphology. Although the complex at this concentration did not significantly interfere in the size of BEAS-2B cells, an increase in the nuclear area of ​​this cell type could be observed, as shown in Fig. 6C. On the other hand, treatment with hmxbato at the highest concentration (19 µM) promoted significant changes in the morphology and pattern of actin polymerization of BEAS-2B cells, significantly decreasing the cell and size of the nucleus. Our results show that hmxbato (3.8 µM) causes significant changes in the morphology of lung tumor cells and cytoskeleton damage, while the same concentration of the complex did not cause major changes in the morphology or cytoskeleton of normal lung cells.

### Hmxbato significantly increases ROS generation in A549 tumor cells

**Figure 7:**
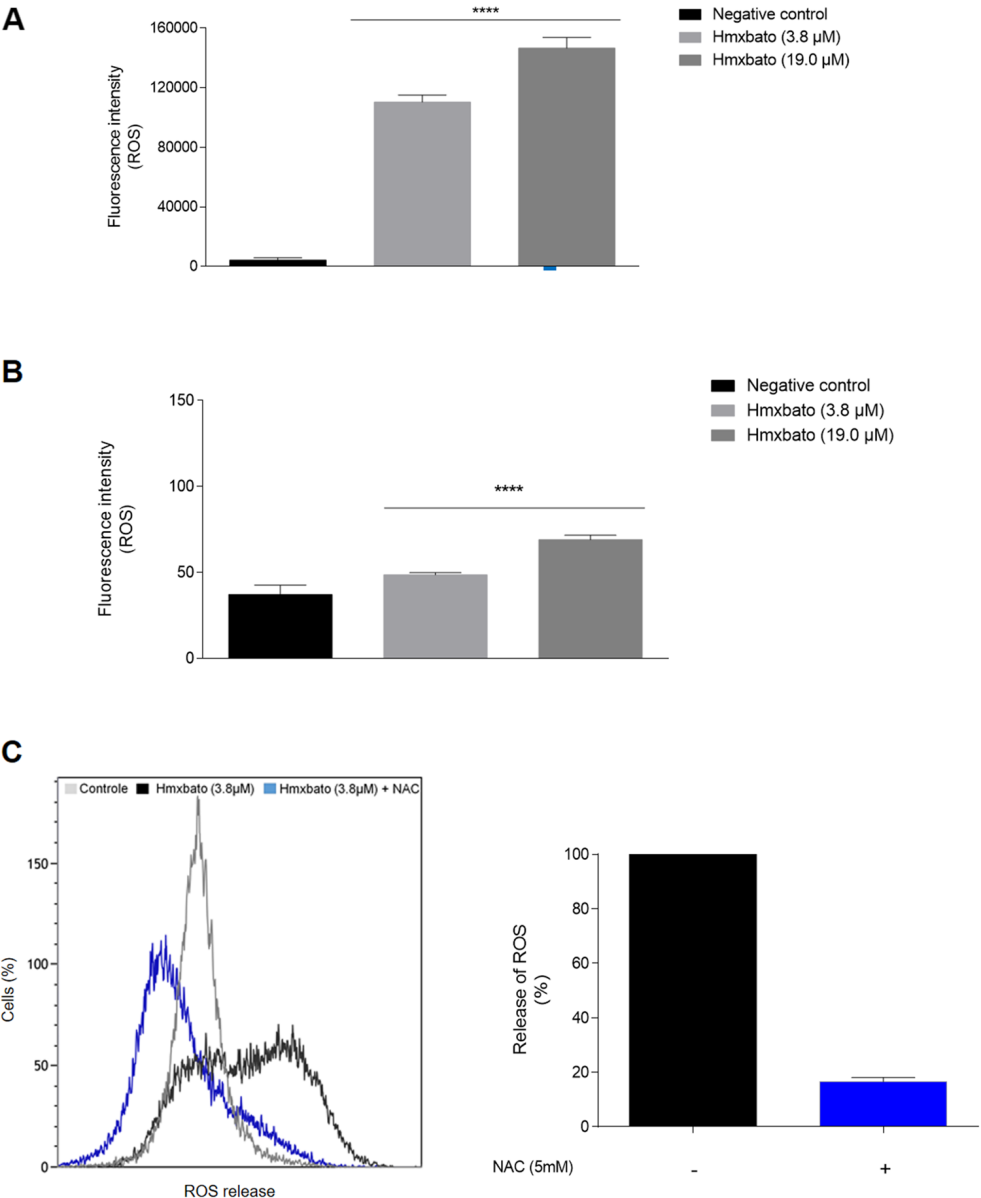
Hmxbato increased reactive oxygen species (ROS) levels in lung tumor cells. Tumor (A549) and normal (BEAS-2B) lung cells were cultured for 4 h in the absence (negative control) or presence of hmxbato (3.8 µM and 19 µM) and subjected to the CM-H_2_DCFDA incorporation assay. Bar graphs show the fluorescence intensity (ROS-positive cells) quantification of A549 (**A**) and BEAS-2B (**B**) cells at the different concentrations tested. Alternatively, the cells were incubated with hmxbato in the presence of NAC and were evaluated using a flow cytometer. (**C**) The histogram shows the release of ROS in control cells (gray line), cells treated only with 3.8 µM hmxbato (black line) and cells treated with 3.8 µM hmxbato in the presence of NAC (blue line). The measures obtained by flow cytometry were used to quantify the data shown in the graph. The graph shows the percentages of ROS release that were calculated considering the treatment with hmxbato in the absence of NAC as 100% ROS release. Data were expressed as mean ± standard deviation of experiments performed in triplicate. Three independent experiments were performed and over 10,000 cells were analyzed in each replicate. Statistical differences were determined using one-way ANOVA and Tukey's multiple comparison test. Significant differences with respect to control were considered when *p* < 0.1 (*), *p* < 0.01 (**), *p* < 0.001 (***) and *p* < 0.0001 (****).

In order to verify whether the apoptosis-induced cell death caused by hmxbato is related to increased ROS generation, tumor and non-tumor cells were incubated with the CM-H_2_DCFDA fluorescent probe and analyzed by flow cytometry. The complex at both concentrations strongly increased ROS production in A549 tumor cells. As shown in Fig. 7A, when compared to the control, treatment of tumor cells with 3.8 µM of the complex caused a 25-fold increase in fluorescence intensity, while treatment with 19 µM increased the fluorescence intensity by 33-fold. Conversely, treatment of BEAS-2B cells with hmxbato at both concentrations produced less significant ROS generation (Fig. 7B). Compared to the control, the 3.8 µM and 19 µM hmxbato treatments induced increases of 1.3-fold and 1.8-fold in fluorescence intensity, respectively. To confirm the hmxbato ability to increase ROS generation by tumor cells, A549 cells were incubated with 3.8 µM of the complex in the presence of NAC, which is an antioxidant molecule capable of inducing the synthesis of glutathione, thus decreasing the ROS production. The cells treated in absence of NAC were considered as capable of increasing the ROS concentrations in 100%. A549 cells in the presence of NAC only increased this production in 16.5% (Fig. 7C). These data show that NAC reduced the ROS production induced by hmxbato in 83.5%, thus confirming the oxidizing action of this complex on tumor cells and its important role in the production of cell stress.

### Hmxbato causes alterations in the mitochondrial membrane potential (∆Ψ_m_) of A549 tumor cells

**Figure 8:**
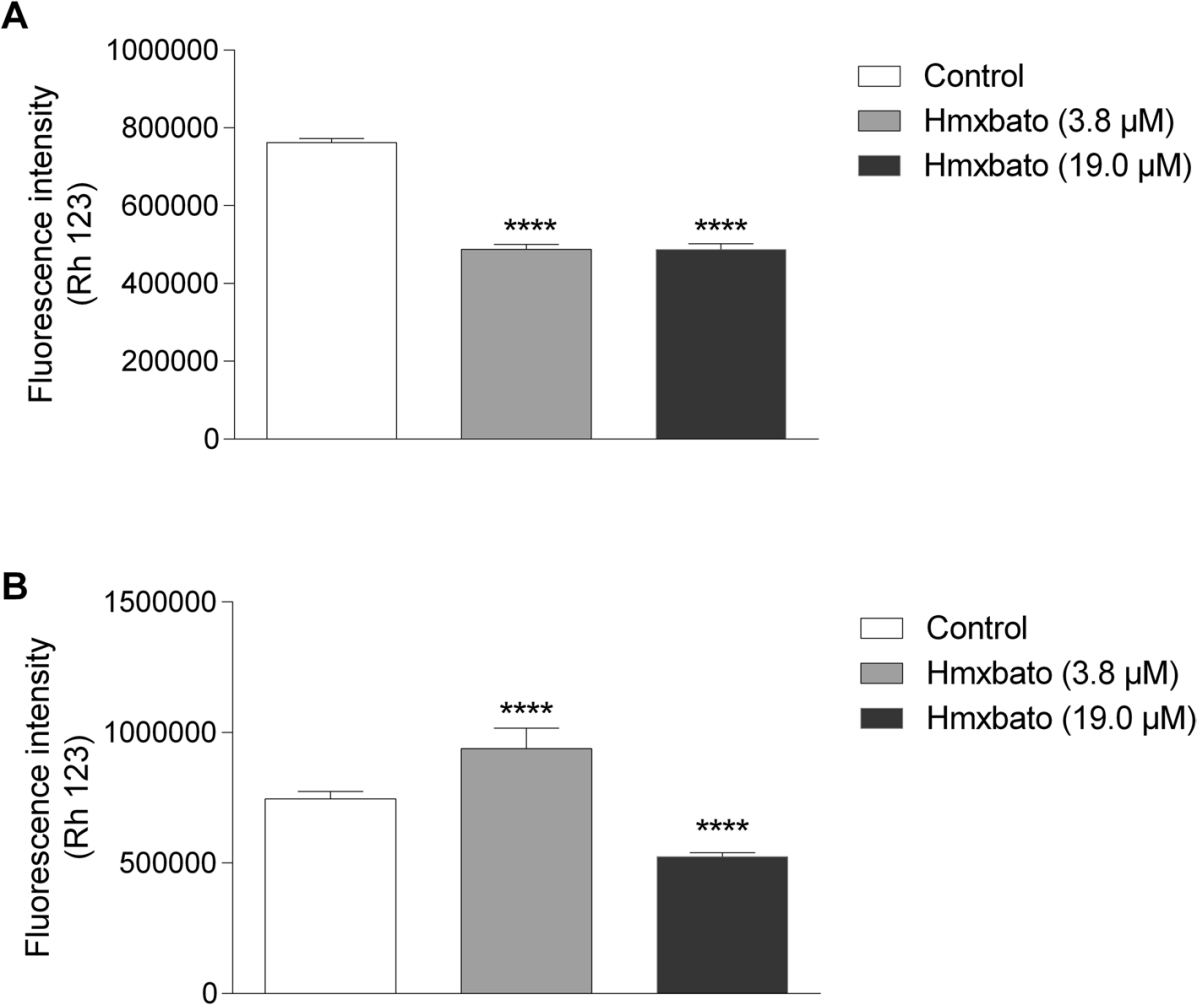
Hmxbato induced mitochondrial damage in lung tumor cells. Tumor (A549) and normal (BEAS-2B) lung cells were incubated for 24 h in the absence (control) or presence of hmxbato (3.8 µM and 19 µM) and stained with rhodamine 123 to detect changes in the mitochondrial membrane potential (∆*Ψ*_*m*_). The fluorescence intensity of A549 (**A**) and BEAS-2B (**B**) cells was quantified by flow cytometry and presented in bar graphs. Data were expressed as mean ± standard deviation of experiments performed in triplicate. Three independent experiments were performed and over 10,000 cells were analyzed in each replicate. Statistical differences were determined using one-way ANOVA and Tukey's multiple comparison test. Significant differences with respect to control were considered when *p* < 0.1 (*), *p* < 0.01 (**), *p* < 0.001 (***) and *p* < 0.0001 (****).

To assess whether hmxbato promotes changes in the mitochondrial membrane potential (∆*Ψ*_*m*_), A549 and BEAS-2B cells treated with different concentrations of the complex were stained with rhodamine 123, a fluorescent probe that accumulates within mitochondria with polarized membranes. Treatment with the complex at both concentrations was able to significantly inhibit the fluorescence intensity of A549 cells, demonstrating its ability to induce ∆*Ψ*_*m*_ depolarization (Fig. 8A). In addition, there were no statistically significant differences between the concentrations tested (3.8 µM and 19 µM). Interestingly, BEAS-2B cells treated with 3.8 µM hmxbato exhibited increased fluorescence intensity, i.e. they presented *∆Ψ*_*m*_ hyperpolarization in relation to control cells (Fig. 8B). In contrast, BEAS-2B cells treated with 19 µM showed a reduction in fluorescence intensity, thus indicating ∆*Ψ*_*m*_ depolarization. These results show that hmxbato (3.8 µM) was able to induce ∆*Ψ*_*m*_ depolarization in lung tumor cells.

### Hmxbato induces genotoxicity in A549 tumor cells

**Figure 9:**
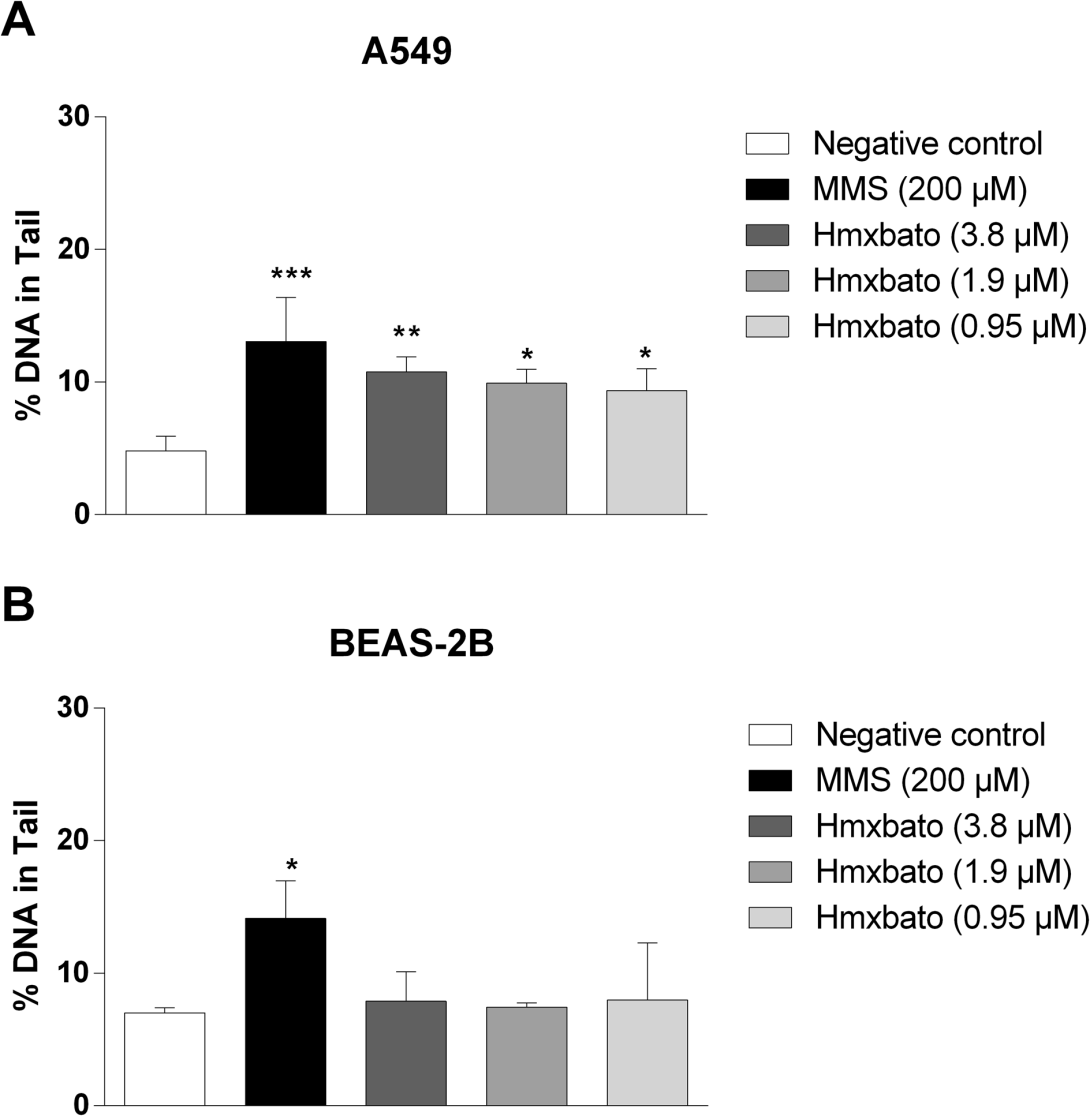
Genotoxic effects of hmxbato on tumor and normal lung cells. Tumor (A549) and normal (BEAS-2B) lung cells were incubated for 4 h with complete medium (negative control), hmxbato (3.8 µM and 19 µM) or methyl methanesulfonate—MMS (200 μM, positive control) and evaluated by the comet assay. Bar graphs show the quantification of the tail DNA percentage in (**A**) A549 and (**B**) BEAS-2B cells, which was used as a parameter for DNA damage. Data were expressed as mean ± standard deviation of experiments performed in triplicate. Three independent experiments were performed and at least one hundred nucleoids were analyzed per sample in each triplicate. Statistical differences were determined using one-way ANOVA and Tukey's multiple comparison test. Significant differences with respect to control were considered when *p* < 0.1 (*), *p* < 0.01 (**), *p* < 0.001 (***) and *p* < 0.0001 (****).

To investigate whether hmxbato promotes genotoxic effects, the comet assay was performed on A549 and BEAS-2B cells after treatment with the complex for 4 h (Fig. 9). The following concentrations were used: 0.25 × IC_50_ (0.95 µM), 0.5 × IC_50_ (1.9 µM) and 1 × IC_50_ (3.8 µM). In addition, methyl methanesulfonate (MMS—200 μM) was used as a positive control for genotoxic damage. After 4 h of treatment, hmxbato induced a statistically significant DNA damage in A549 cells at all concentrations tested (Fig. 9A). In BEAS-2B cells, hmxbato did not promote DNA damage at any of the concentrations evaluated (Fig. 9B). As expected, MMS promoted genotoxic effects on the DNA from both A549 and BEAS-2B cells. These results corroborate our aforementioned findings, showing that hmxbato exhibits greater action selectivity on tumor cells than on normal lung cells.

### Hmxbato increases caspase-9 activity in A549 tumor cells

**Figure 10:**
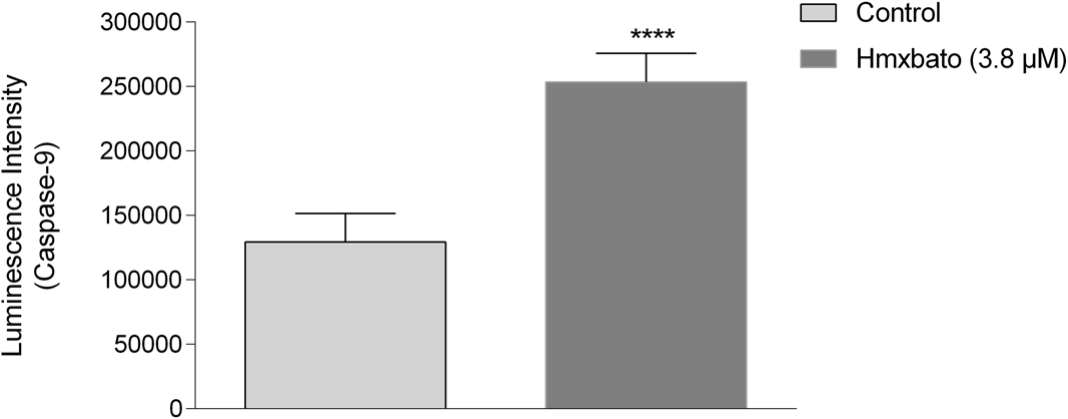
Effects of hmxbato on the caspase-9 activity in lung tumor cells. A549 cells were treated with 3.8 µM of hmxbato and detection of the caspase-9 activity was assessed using the Caspase-Glo 9 Assay kit (Promega). The graph shows the luminescence values ​​obtained in a microplate reader, processed and analyzed using the GraphPad Prism software version 6.01. Data were expressed as mean ± standard deviation of experiments performed in triplicate. Three independent experiments were performed. Statistical differences were determined using one-way ANOVA and Tukey's multiple comparison test. Significant differences with respect to control were considered when *p* < 0.1 (*), *p* < 0.01 (**), *p* < 0.001 (***) and *p* < 0.0001 (****).

Considering the antitumor potential and mitochondrial membrane depolarization induced by the hmxbato complex in A549 tumor cells, we further investigated the influence of this complex on the intrinsic apoptotic pathway with the *Caspase-Glo 9 Assay Kit* (Promega). As shown in Fig. 10, the hmxbato complex promoted a statistically significant induction of the caspase-9 activity in A549 cells, with a 1.96-fold increase when compared to the control. This result supports the involvement of hmxbato in the apoptosis activation in A549 tumor cells through the intrinsic pathway.

### Hmxbato increases the expression of caspase 3 and PARP-1

**Figure 11:**
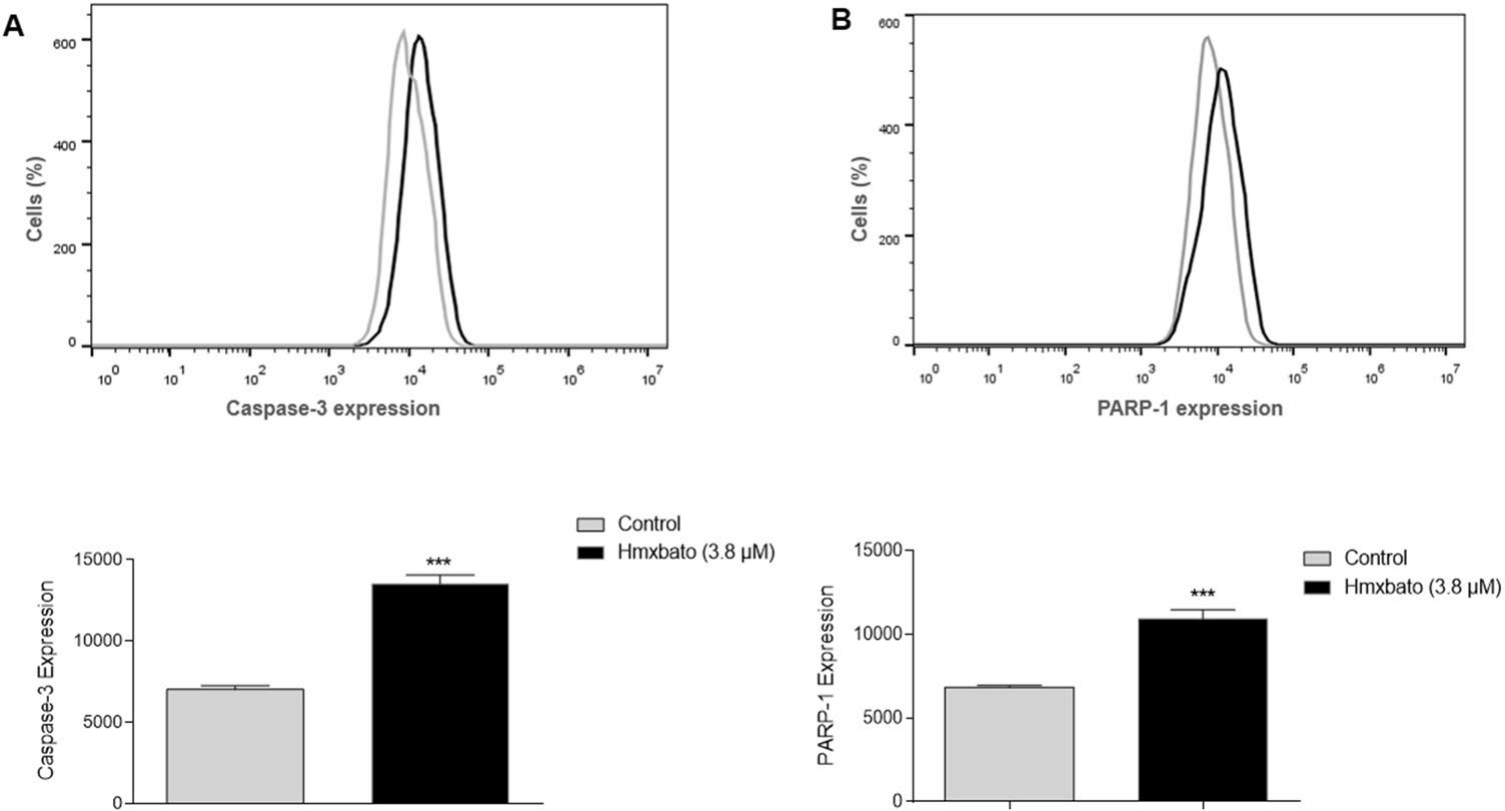
Effects of hmxbato on the expression of caspase-3 and PARP-1 in lung tumor cells. A549 cells treated with 3.8 µM hmxbato were assayed by the CytoFix / Cyto Perm kit (BD Pharmingen) to determine the caspase-3 and PARP-1 expression levels. The histograms show (**A**) caspase-3 and (**B**) PARP-1 expressions in A549 cells. The bar graphs show the median values for expressions after analysis by GraphPad Prism Software version 6.01. Data were expressed as mean ± standard deviation of experiments performed in triplicate. Three independent experiments were performed. Statistical differences were determined using one-way ANOVA and Tukey's multiple comparison test. Significant differences with respect to control were considered when *p* < 0.1 (*), *p* < 0.01 (**), *p* < 0.001 (***) and *p* < 0.0001 (****).

In order to confirm the involvement of the hmxbato complex in the caspase pathway and consequent activation of apoptosis, the expression of the executioner caspase 3 was evaluated using the Cyto Fix / Cyto Perm Kit (BD Pharmingen). As shown in Fig. 11A, hmxbato promoted a statistically significant increase in caspase-3 expression, an 1.92-fold increase when compared to the control. Also, it is worth mentioning that caspase-3 was able to cleave and activate PARP-1, a nuclear enzyme that plays a central role in DNA repair. In Fig. 11B, a 1.59-fold increase in the PARP-1 enzyme expression in A549 cells treated with the hmxbato complex was observed, which is a statistically significant increase when compared to the control. Together, these results further reinforce the hypothesis that the hmxbato complex can promote apoptosis by activating the caspase pathway, especially through the intrinsic pathway, and it is also capable of inducing damage to cell DNA through activation of the PARP-1 enzyme.

### DNA docking studies of hmxbato

**Figure 12:**
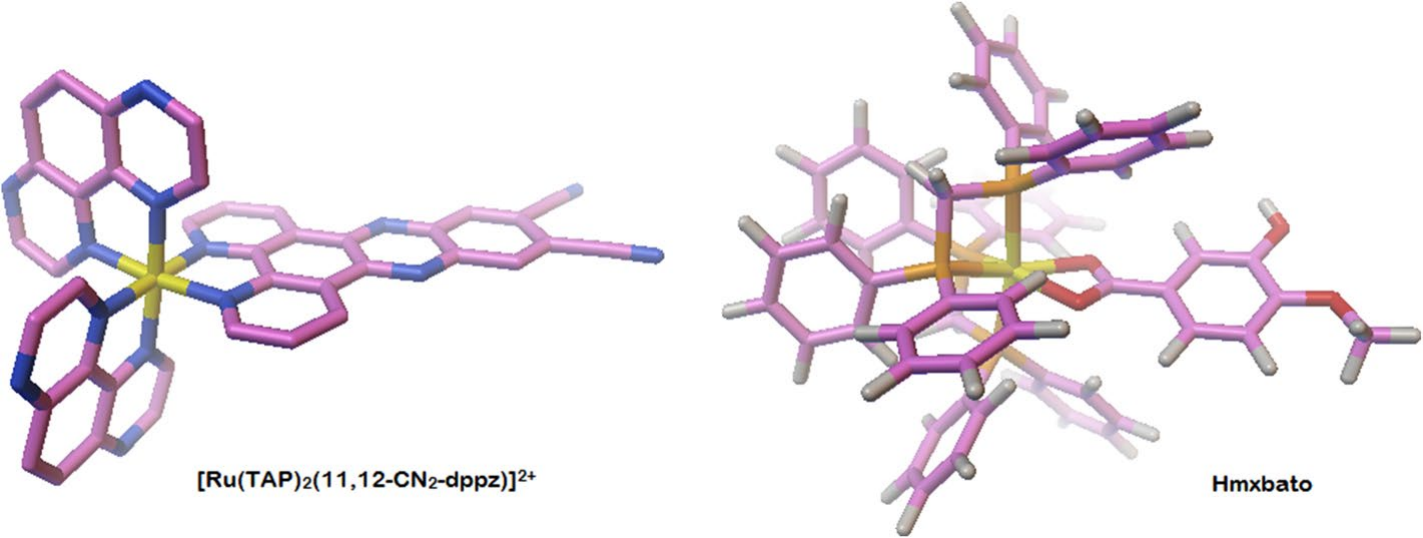
Structure of the [Ru(TAP)_2_(11,12-CN_2_-dppz)]^2+^ (left) and hmxbato (right) ligands using the AutoDock 4.0 program.

**Figure 13:**
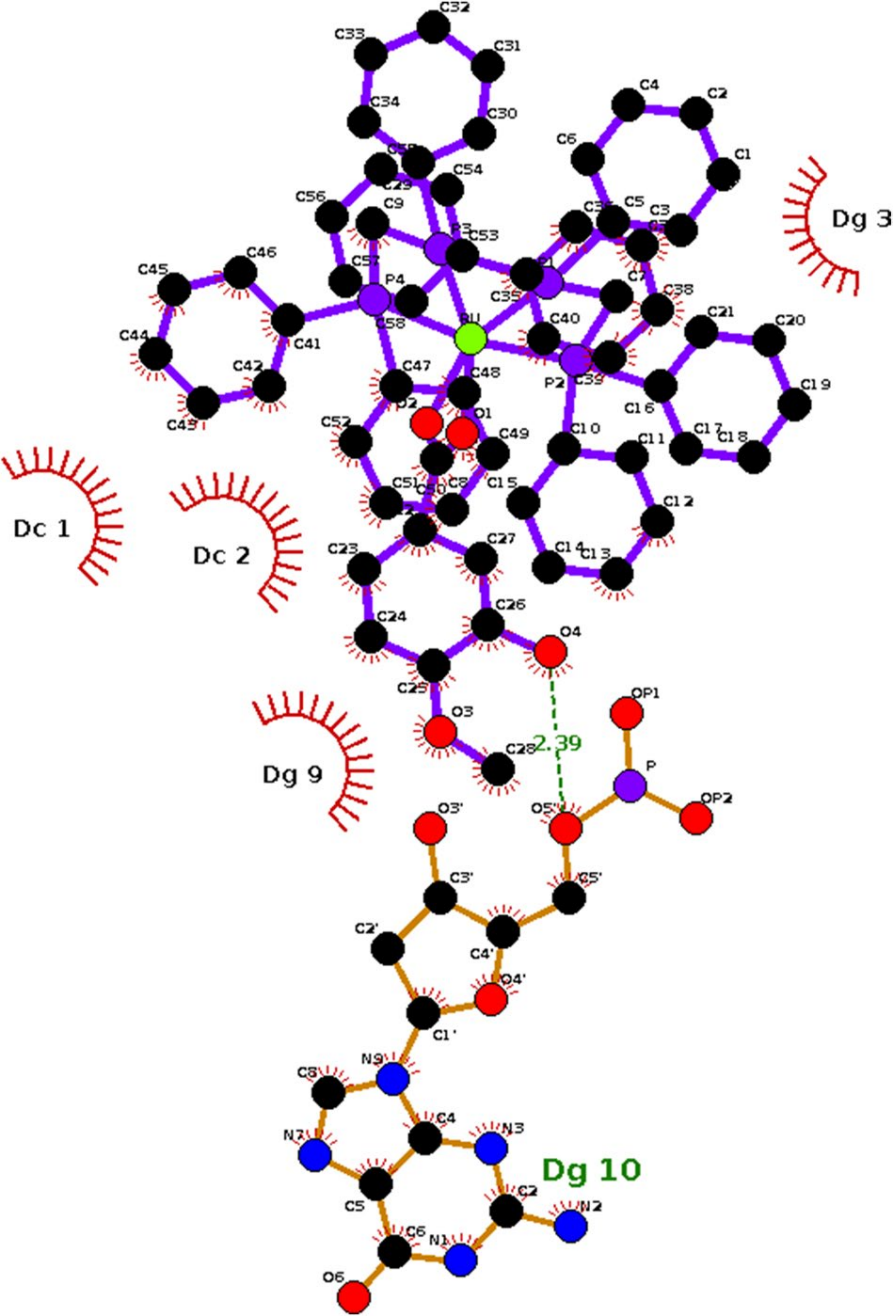
Identification of the intermolecular interactions between the hmxbato ligand and DNA, using the LigPlot program^30^.

From the theoretical studies of molecular docking performed in the AutoDock 4.0 program for DNA (PDB ID: 6G8S), the ten conformations with the best binding energies were evaluated for both studied complexes. The conformation chosen was the one that had the lowest RMSD (Root Mean Square Deviation) and the most favorable binding free energy (∆*G*_*binding*_), torsional free energy (∆*G*_*torsional*_) and inhibition constant (*Ki*) values (Table 2). It should be noted that the first system analyzed was used in the re-docking procedure, which consists in validating whether the docking methodology used can calculate the binding energy between ruthenium complexes and DNA. Considering Table 2, the Λ-[Ru(TAP)_2_(11,12-CN_2_-dppz)]^2+^ ligand showed binding energy of − 10.17 kcal mol^−1^ and inhibition constant in the nanomolar order, which are expected values for drugs with high efficiency of interaction with their targets^18–21^. Thus, considering that the ligand-DNA complex has RMSD of 0.499 Å compared to its complexed structure obtained by X-ray crystallography, it can be said that the methodology was validated for the characterization of the interaction of Ru + DNA complexes^22^. Regarding hmxbato, interaction energy and an inhibition constant favorable to the formation of the target-ligand complex were observed. This suggests that the compound in question is very likely to have activity. However, the difference between the binding free energies of the analyzed ligands was 3.25 kcal mol^−1^ less favorable for hmxbato than for the reference ligand Λ-[Ru(TAP)_2_(11,12-CN_2_-dppz)]^2+^. This difference is expected because the reference compound is expressively flatter than hmxbato (Fig. 12), which can be evidenced by the difference of 2.47 kcal mol^−1^ in the torsional energy between the two compounds (Table 2). Thus, the 3.02 kcal mol^−1^ torsional energy of hmxbato is justified by the greater degree of freedom of this compound, which resulted in a decrease in the binding energy of this magnitude and, consequently, in an inhibition constant value approximately 224 times higher in comparison to the reference ligand, which has a more favorable interaction free energy since it presents a better fit between the DNA double helix and the flat structure of the compound Λ-[Ru(TAP)_2_(11,12-CN_2_-dppz)]^2+^^21,23^. Based on the hypothesis that interaction between DNA and hmxbato occurs, the LigPlot program^24^ was used to understand the nature of those interactions. The results suggest that hmxbato interacts with DNA through hydrophobic interactions between guanines (DG3 and DG9) and cytosines (DC1 and DC2) of DNA and aromatic rings of hmxbato, and it remains bound to DNA by hydrogen bonding between the hydrogen atom of the -OH group of hmxbato and an oxygen (phosphate) of one of the guanines of the DNA structure (Fig. 13)^18,25^.

**Table 2:** Estimates of binding free energy, torsional energy and their inhibition constants for both DNA complexes, code 6G8S.

Ligands	DNA		
	*∆G*_*binding*_ (kcal mol^−1^)	*∆G*_*torsional*_ (kcal mol^−1^)	*K*_*i*_ (μM)
[Ru(TAP)_2_(11,12-CN_2_-dppz)]^2+^	− 10.17	+ 0.55	0.035
Hmxbato	− 6.96	+ 3.02	7.850

## Discussion

Ruthenium complexes have been extensively explored in recent years mainly due to their antitumor^26–29^ and antiparasitic activities. Some studies of our research group have investigated the cytotoxic potential of different ruthenium complexes against parasites of the *Leishmania* genus^10,30^ (Costa et al., 2017; Miranda et al., 2018), and we have also demonstrated the probable cell death mechanism triggered by ruthenium complex called 3-hydroxy-4-methoxybenzoate (hmxbato)-*cis*-[Ru^II^(ŋ^2^-O_2_CC_7_H_7_O_2_)(dppm)_2_]PF_6_ against *Leishmania (Leishmania) amazonensis* promastigotes^11^. Considering this and the significant anti-*Leishmania* activity of hmxbato^10^, in the present study, we investigated the cytotoxic effects of hmxbato on tumor cells.

Our results showed cytotoxic potential for hmxbato as well as its selective toxicity against lung tumor cells from the A549 cell line. Previous studies have demonstrated the cytotoxic activity of ruthenium complexes against different tumor cell lines. Complexes 1b and 2b containing phenylterpyridine (phtpy) derivatives displayed higher cytotoxicity on A549 cells after 72 h of treatment, showing IC_50_ values of 31.7 µM and 29.8 µM, respectively^31^. More recently, three ruthenium complexes containing salicylate as ligand, [Ru(phen)_2_(SA)] (phen = 1,10-phenanthroline, SA = salicylate, **1**), [Ru(dmb)_2_(SA)] (dmb = 4,4′-dimethyl-2,2′-bipyridine, **2**) and [Ru(bpy)_2_(SA)] (bpy = 2,2′-bipyridine, **3**), were analyzed for their cytotoxic potential against lung tumor and non-tumor cells. Complexes **1**, **2** and **3** exhibited cytotoxic activity against A549 cells after 48 h of treatment, with IC_50_ values of 17.7 µM, 28.1 µM and 40.5 µM, respectively, while the treatment of BEAS-2B cells showed IC_50_ values of 62.5 µM, 50.1 and 64.3 µM, respectively^32^. The cytotoxic potential of phosphine/diimine ruthenium complexes against A549 tumor cells was demonstrated after 24 h of treatment with the complexes [Ru(law)(N–N)_2_]PF_6_, where N–N is 2,2′-bipyridine **(1)** or 1,10- phenanthroline **(2),** and [Ru(law)(dppm)(N–N)]PF_6_, where dppm means bis(diphenylphosphino)methane and N–N is 2,2′-bipyridine **(3)** or 1,10-phenanthroline **(4)**, resulting in IC_50_ values of 99.1 µM, 69.1 µM, 4.8 µM and 4.2 µM, respectively^33^. Thus, our results for hmxbato highlight its excellent cytotoxic potential against lung tumor cells, since a lower IC_50_ value (3.8 µM) was found even after 24 h of treatment. In addition, it is important to note that hmxbato remains stable in DMSO, showing no structural changes during the 72 h analysis time (supplementary data).

A very important property that must be taken into consideration for the development of a new anticancer drug is its selectivity of action, i.e. the ability of the drug to promote selective toxicity on tumor cells^34^. This property can be assessed through the selective cytotoxicity index (ICS), calculated as the ratio of the IC_50_ values for normal and tumor cells. As a result, ICS values ≤ 1 indicate a lack of selective cytotoxicity, while values > 1 indicate selective cytotoxicity. The hmxbato showed an excellent ICS (> 10), indicating that this complex presents preferential cytotoxic action on lung tumor cells. Aiming at its potential as an anticancer molecule, this characteristic makes the hmxbato complex even more interesting.

Reinforcing the idea of selectivity, it was also demonstrated that hmxbato significantly inhibits the proliferation and recovery of A549 cells at all concentrations tested, except for the recovery of cells treated with 0.95 µM hmxbato after 48 h of incubation. This same effect was not observed in BEAS-2B cells, since concentrations of 0.95 µM, 1.9 µM and 3.8 µM did not interfere with normal cell proliferation in the initial times. Taken together, these results demonstrate that hmxbato at a concentration of 3.8 µM is highly selective and cytotoxic to lung tumor cells. Furthermore, the hmxbato complex had a negative effect on the survival and proliferation of A549 cells and still interfered negatively in the formation of colonies, since no cells were able to grow and form colonies even in the presence of the lowest concentrations of the complex. In contrast, BEAS-2B cells survived and later formed colonies, except for cells treated with the highest concentration (19 µM) of the complex. Other studies using ruthenium complexes and A549 cells also demonstrated this interference in colony formation^29,35^, but the concentrations of the complexes required for this effect were higher than those described for hmxbato.

The development of antitumor drugs is linked to the understanding of the mechanisms of action promoted by the candidate molecules. In this sense, the knowledge of molecular and biochemical mechanisms is of great relevance^3^. The probable mechanisms of action of many recently described ruthenium complexes involve apoptosis^27,32,36–38^.

Most apoptotic pathways involve activation of caspases, and the cytoskeleton may be a target for the proteolytic activity of some effector caspases. Thus, morphological changes and alterations in the actin cytoskeleton organization can be seen in apoptotic cells^39–41^. Hmxbato induced significant changes in A549 cell morphology and caused damage to the cytoskeleton. Another modification that may also be associated with cell death by apoptosis is cell cycle arrest^42,43^. We demonstrated that hmxbato was able to promote a strong G2/M phase arrest in lung tumor cells. Chen et al. described this same effect on the cell cycle of A549 cells treated with the polypyridyl complex [Ru(bpy)_2_(pytp)]^2+^ (RU2)^44^. Our results also showed that hmxbato did not promote significant changes in the cell cycle of normal lung cells.

Some chemotherapeutic drugs, such as doxorubicin and cisplatin, induce increased ROS levels, which would contribute to cell toxicity^45,46^. Hmxbato was also able to promote changes in ROS levels of A549 cells, significantly increasing the fluorescence intensity of tumor cells treated with different concentrations of the complex. A recent study using two new polypyridyl ruthenium (II) complexes, called Ru(II)-1 ([Ru(dmp)_2_(CAPIP)](ClO_4_)_2_) and Ru(II)-2 ([Ru(dmp)_2_(CFPIP)](ClO_4_)_2_), also demonstrated increased levels of ROS in A549 cells. In this case, the complexes at different concentrations caused increases of 40.3 (4.0 µM Ru(II)-1), 17.4 (2.0 µM Ru(II)-1), 44.7 (6.0 µM Ru(II)-2) and 20.3 (3.0 µM Ru(II)-2) times the fluorescence intensity of tumor cells relative to the control^43^. Complementarily, we demonstrated that hmxbato promoted a slight increase in ROS levels of non-tumor cells. Nevertheless, it should be noted that the increase in fluorescence intensity of BEAS-2B cells treated with 3.8 µM hmxbato was significantly lower when compared to the increase detected for A549 tumor cells. In addition, the ability of hmxbato to act as an oxidizing molecule, inducing overproduction of ROS in tumor cells was confirmed by incubating the complex with the NAC inhibitor. As a result, there was a significant decrease in the ROS formation in cells.

ROS are considered essential in cell homeostasis and can perform important functions such as (1) regulation of cell cycle progression and proliferation; (2) regulation of differentiation, migration and cell death; (3) maintenance of redox balance and (4) immune response interference. However, high levels can induce cell stress, causing harmful effects that can lead to cell death^41,47,48^.

Our results showed that hmxbato induced A549 cell death by apoptosis in approximately half of the treated cells. Once again, this corroborate other studies described in the literature that also showed A549 cell death by apoptosis after treatment with different ruthenium complexes^29,32,35,49^. We further demonstrated that 3.8 µM hmxbato only induced a small percentage of death in non-tumor cells, maintaining more than 85% of viable cells. This difference in the survival of tumor and non-tumor cells treated with hmxbato may be explained by the fact that tumor cells have higher basal levels of ROS generation in comparison to those of non-tumor cells. Therefore, tumor cells would have a lower redox homeostasis tolerance threshold than non-tumor cells, thus making tumor cells more prone to cell death^50,51^. This justifies the results obtained for 3.8 µM hmxbato and the slight increase in ROS production in BEAS-2B cells, associated with the survival of these cells.

Mitochondria is the main organelle producing ROS and an imbalance between antioxidant defenses and the production of reactive oxygen species can cause cell damage. In this context, mitochondria play a key role in programmed cell death^52–54^. Induced alterations in mitochondrial potential can decrease cell viability^55,56^. Our results showed that hmxbato promoted depolarization of the mitochondrial membrane potential of A549 cells treated with both concentrations of the complex. In addition, 3.8 µM hmxbato induced mitochondrial hyperpolarization in BEAS-2B cells. When hyperpolarization is not accompanied by excessive ROS generation, it may have a signaling function capable of alleviating damages that could be produced by a subsequent intense ROS release^55,56^, thus justifying the mitochondrial hyperpolarization without impaired cell survival detected in BEAS-2B cells after treatment with 3.8 µM hmxbato.

Studies have shown that mitochondrial depolarization can lead to the release of the soluble hemeprotein cytochrome c, activating caspases and inducing the apoptosis process^54,56^. Hmxbato increased the caspase-9 activity in A549 cells. This initiator caspase is involved in the process of activating the intrinsic apoptosis that occurs via mitochondria. In addition, we also demonstrated that hmxbato increased the expression of executioner caspase 3 in tumor cells. Together, these results suggest that hmxbato induces an overproduction of ROS, thus causing an imbalance in the redox cell homeostasis and consequent alteration in mitochondrial membrane potential. In this sense, changes in the mitochondrial potential can induce the activation of caspase-9 (initiating caspase) and then of caspase-3 (executioner caspase), thus promoting cell death by apoptosis.

Cellular stress can promote the activation of regulatory molecules from repair systems. Among them, we can highlight the nuclear enzyme PARP-1, which plays a central role in DNA repair. This nuclear enzyme can be activated by some executioner caspases such as caspase-3 and caspase-7^57–60^. The hmxbato complex was able to increase the expression of the PARP-1 enzyme in A549 cells, indicating its possible interference in the DNA of cells.

**Figure 14:**
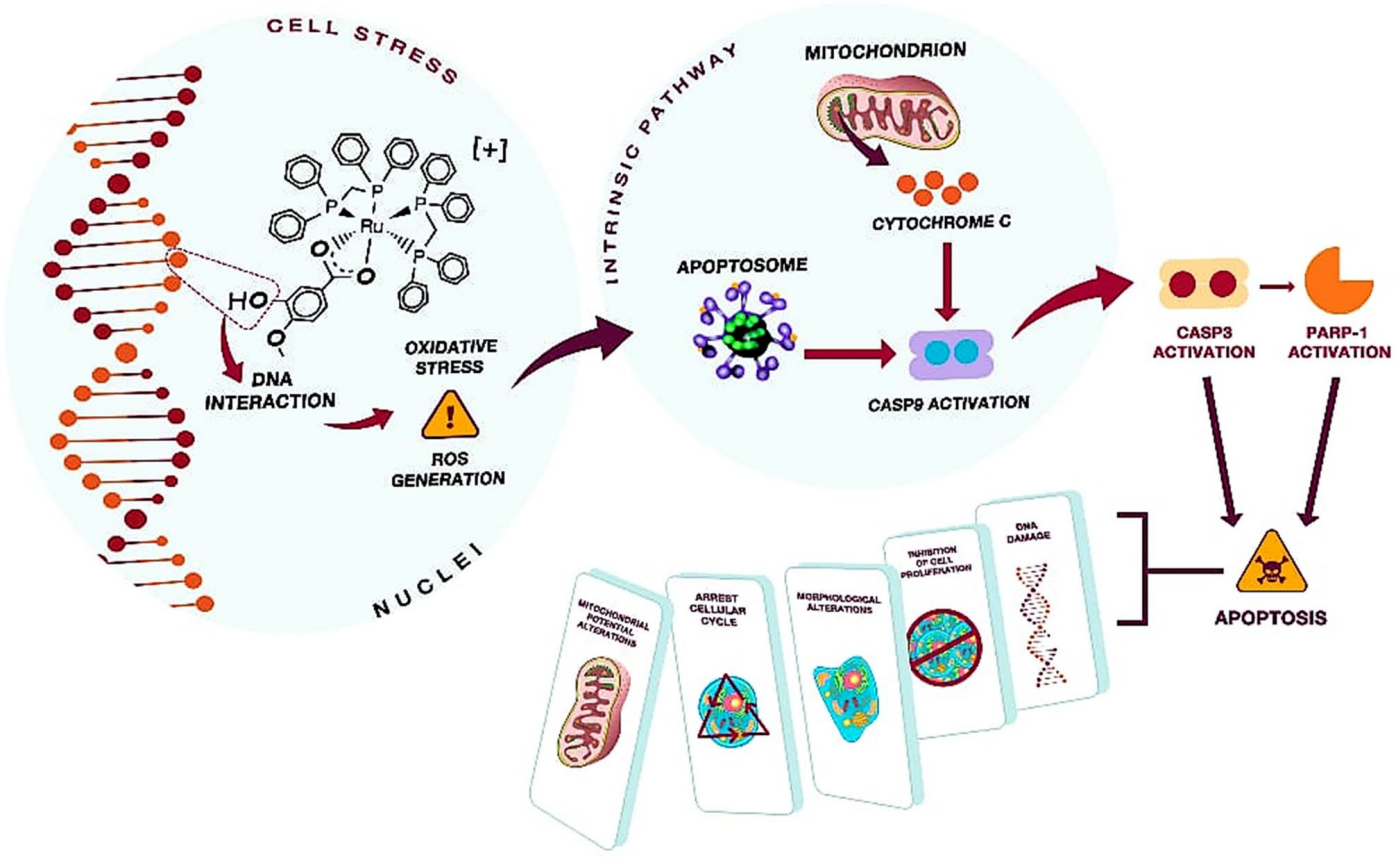
Proposed mechanism of action for the antitumor activity of hmxbato against A549 lung tumor cells. The interaction of hmxbato with cell DNA causes an oxidative stress which results in increased ROS production. The ROS overproduction induces the activation of the intrinsic apoptotic pathway due to changes in the mitochondrial potential. The mitochondrial damage results in cytochrome C release in the cytosol with subsequent caspase-9 activation. The caspase-9 promotes the caspase-3 activation, which will activate the enzyme PARP-1. Finally, the cell death is induced by apoptosis, justifying the following cellular events: (1) changes in mitochondrial potential; (2) cell cycle arrest; (3) morphological changes; (4) inhibition of cell proliferation and (5) DNA damage.

In addition, as demonstrated by the comet assay, hmxbato caused DNA damage in A549 cells after 4 h of treatment. No genotoxic effects were detected for BEAS-2B non-tumor cells, reinforcing the selectivity of hmxbato for tumor cells. Finally, molecular docking studies suggested that the DNA is a possible biological target for hmxbato, with theorical values of *∆G*_*binding*_ e *K*_*i*_ favorable for hmxbato-DNA system formation. The interactions involved are hydrophobic between guanines (DG3 and DG9) and cytosines (DC1 and DC2) of DNA and aromatic rings of hmxbato, in addition to the hydrogen bond between the hydrogen atom of the -OH group of the hmxbato and the oxygen atom (phosphate) from guanine DG10. This interaction may be responsible for signaling the DNA damage, which would cause oxidative stress in the cell and subsequent significant increases in ROS levels, leading to alterations in the mitochondrial membrane potential, cell cycle arrest, morphological changes, inhibition of cell proliferation and DNA damage followed by apoptosis (Fig. 14).

Other studies using ruthenium complexes have demonstrated its cytotoxic potential against different types of tumors^17,61–63^ and the DNA has been the target of these different complexes, which may culminate in cell death by apoptosis^37,43,50,64^. In this sense, it is worth mentioning that in addition to promoting apoptosis, hmxbato presented selective cytotoxicity for tumor cells, thus ensuring the survival of normal lung cells at the concentration of 3.8 µM. This important feature makes hmxbato an interesting molecule for prospecting new therapies for lung cancer.

In conclusion, our original study describes the cytotoxic potential of a ruthenium complex (hmxbato), as well as its action selectivity for lung tumor cells. Additionally, we found that this complex at a concentration of 3.8 µM promotes significant increased ROS levels in tumor cells, generating oxidative stress and culminating in: (1) reduction of cell proliferation; (2) changes in cell morphology and organization patterns of the actin cytoskeleton; (3) cell arrest in the G2/M phase of the cell cycle; (4) apoptosis; (5) changes in the mitochondrial membrane potential and (6) DNA damage. In addition, we demonstrated that this same concentration of hmxbato ensures the survival of most normal lung cells. Furthermore, we demonstrated that the induction of programmed cell death can occur by the intrinsic apoptotic pathway through the activation of caspases. It should also be emphasized that the selective action of hmxbato on tumor cells is an uncommon property for most existing chemotherapeutic agents since most of them also exhibit high toxicity on non-tumor cells. Thus, this important feature makes this ruthenium complex an interesting candidate for the design of new drugs against lung cancer. In our future studies, we will investigate the genes involved in the apoptotic pathway, as well as the pharmacokinetic properties of hmxbato.

## Methods

### Reagents

Cisplatin-Pt(NH_3_)_2_Cl_2_), hydrogen peroxide solution (H_2_O_2_), dimethylsulfoxide (DMSO), penicillin, streptomycin, rhodamine 123, methyl methanesulfonate (MMS), Dulbecco's Modified Eagle Medium: Nutrient Mixture F-12 medium (DMEM/F12), were purchased from Sigma Chemical Co. (USA), annexin V FITC apoptosis detection kit from BD Pharmingen and heat-inactivated fetal bovine serum (FBS) from Cultilab (Brazil). All other reagents were analytical grade or superior.

### Synthesis of ruthenium(II) complexes

The precursor complex (cis‑[Ru^II^(η^2^-O_2_CR)(dppm)_2_]PF_6_) was synthesized as described in the literature^65^ and hmxbato (cis‑[Ru^II^(ŋ^2^-O_2_CC_7_H_7_O_2_)(dppm)_2_]PF_6_) was synthesized and purified as previously described^10^. Additionally, the synthetic route of the hmxbato complex was illustrated in Fig. 1. The complexes were solubilized in dimethyl sulfoxide (DMSO) to obtain a 10 mM stock solution of complex and stored at 4 °C. For the experiments, new dilutions were prepared in culture medium to ensure that the DMSO concentration did not exceed 0.1% in culture medium. The stability of the complex hmxbato in DMSO was monitored by ^31^P {^1^H} NMR experiments for a 72 h period using 1.0 × 10^−3^ mol L^−1^ solution of the complex (supplementary data).

### Cellular culture

The tumor cell lines A549 (adenocarcinomic human alveolar basal epithelial cells) and normal BEAS-2B (human bronchial epithelial cells) were obtained from American Type Culture Collection (ATCC, Manassas,VA). All cells lines were routinely maintained in Dulbecco's Modified Eagle Medium: Nutrient Mixture F-12 medium (DMEM/F12) supplemented with 10% fetal bovine serum (FBS), 1% penicillin (100 UI mL^−1^) and streptomycin (100 µg mL^−1^)—complete DMEM/F12 and incubated at 37 °C in a humidified 5% CO_2_ incubator.

### In vitro cytotoxicity assay

Briefly, A549 and BEAS-2B cells were plated (2 × 10^4^cells/well) in 96-well culture plates containing complete DMEM/F12 medium and incubated at 37 °C in a 5% CO_2_ incubator for 12 h. Then, cells were incubated with complete DMEM/F12 medium in the absence (control) or presence of different concentrations (twofold serial dilution from 200 μM to 0.097 μM) of hmxbato (precursor or ligand-free) for 24 h. Alternatively, cells were also treated with 0.1% DMSO (data not shown). Cisplatin was used as a positive control. The cell viabilities were assessed by the MTT assay according to Mosmann^66^ with modifications. The 50% inhibitory concentration (IC_50_) for non-tumor cells and tumor cells were determined by non-linear regression using the GraphPad Prism Software version 6.01 (GraphPad Software Inc., San Diego, USA). Additionally, the selective cytotoxicity index (ICS) was defined by the ratio between the IC_50_ of non-tumor cells (BEAS-2B) and that of tumor cells (A549)^61^. The SCI indicates the efficiency of a drug to promote death of tumor cells with minimal toxicity to non-tumor cells. SCI values greater than 1 indicate a selective cytotoxicity of the hmxbato complex towards tumor cells. Data were presented as the mean ± standard deviations (SD) and the experiments were performed in quadruplicate and three independent experiments were conducted.

### Cell proliferation assay and recovery of cell proliferation

In order to evaluate the effects of hmxbato on the proliferation of tumor and normal lung cells, 2 × 10^4^ cells/well of A549 and BEAS-2B were cultured in 96-well plates containing complete DMEM/F12 and incubated at 37 °C in a 5% CO_2_ incubator for 12 h. Next, the cells were incubated with complete DMEM/F12 in the absence (control) or presence of different concentrations of hmxbato (0.95, 1.9, 3.8, 19.0 and 38.0 μM) for 24, 48 e 72 h. The cell proliferation assay was measured by MTT as previously described and results were expressed as absorbance values at 570 nm. The experiments were performed in triplicate and three independent experiments were conducted.

Alternatively, the recovery of cell proliferation assay was performed. Here, A549 and BEAS-2B cells were seeded at a density of 2 × 10^4^ cells/well in 96-well plates. After adhesion (12 h), cells were incubated with complete DMEM/F12 (control), DMEM/F12-FBS free or different concentrations of hmxbato (0.95, 1.9, 3.8, 19.0 and 38.0 μM) for 24 h. Thereafter, the treatment or controls were removed and culture medium containing 10% FBS (complete DMEM/F12) was added in all plate wells. Then, recovery of cell proliferation was evaluated at 0, 24 and 48 h. The experiments were performed in triplicate and three independent experiments were conducted.

### Clonogenic assay

As previously reported^67^, the clonogenic assay was performed with some modifications. Briefly, A549 and BEAS-2B (2 × 10^4^cells/well) cells were plated in 96-well culture plates containing complete DMEM/F12 and incubated at 37 °C in a 5% CO_2_ incubator for 12 h. Next, the cells were incubated with complete DMEM/F12 medium in the absence (control) or presence of hmxbato (1.9, 3.8 and 19 μM) for 24 h. In sequence, the cells were collected by enzymatic digestion (Trypsin / EDTA), centrifuged at 1500 rpm for 5 min at 4 °C and seeded (100 cells/well) in 6-well plates containing complete DMEM/F12 medium for 14 days. Finally, the colonies were fixed and stained with crystal violet. Only the colonies with ˃50 cells were counted by direct visual inspection. The experiments were performed in triplicate and three independent experiments were conducted. The data are presented as the mean ± SD.

### Cell cycle analysis

The distribution of cell cycle was carried out as described previously^68^ and analyzed by flow cytometry. A549 and BEAS-2B (1 × 10^6^ cells mL^−1^) cells were placed into a 6-well plate and incubated at 37 °C in a 5% CO_2_ for 12 h. Subsequently, the cells were incubated for 24 h in the absence (control) or presence of hmxbato (3.8 and 19.0 μM). After incubation, cells were washed in PBS and resuspended in a solution of 70% ice-cold ethanol in PBS and fixed for 18 h at 4 °C. Subsequently, the cells were incubated with 10 µg mL^−1^ propidium iodide and 100 µg mL^−1^ RNAse A in PBS for 45 min at 37 °C in the dark. The cell population analyses were performed with a flow cytometer (CytoFLEX Platform—Beckman Coulter) and more than 10,000 events were acquired. The software Kaluza was used to analyze the percentages of cells in each cell cycle phase: G1, S and G2-M. The experiments were performed in triplicate and three independent experiments were conducted. The data are presented as the mean ± SD.

### Evaluation of apoptosis

A549 and BEAS-2B cells (3 × 10^5^/well) were incubated in 12-well plates at 37 °C in a 5% CO_2_ for 12 h and were treated or not (control) with 3.8 and 19.0 μM of hmxbato for 24 h. Subsequently, the cells were incubated with a specific binding buffer containing annexin V-FITC and propidium iodide (BD Bioscience, Brazil) for 15 min at 25 °C, in the dark, according to manufacturer’s instructions. Immediately, cells were analyzed by flow cytometer (CytoFLEX Platform—Beckman Coulter) and more than 10,000 events were acquired. Cells treated with cisplatin (400 µM) or formaldehyde (4%) were used as internal controls for apoptosis and necrosis, respectively. The Kaluza software was used for the analysis of results and the quantitative graphs represent the average values obtained in independent experiments. The total percentage of cells in apoptosis was considered as the sum of both early and late apoptosis (annexin V-FITC positive), lower and upper right quadrants in the two-parameter flow cytometric dot plots, as previously described^17^.

### Cellular morphology analysis

As previously reported^11^, the cellular morphology analysis assay was performed with some modifications. To evaluate the effect of the complex on the morphology of normal and tumor lung cells, they were stained with Alexa Fluor 488 phalloidin. A549 and BEAS-2B (1 × 10^5^/well) were incubated in 24-well plates at 37 °C in a 5% CO_2_ for 12 h and were treated or no (control) with 3.8 and 19.0 μM of hmxbato for 24 h. The cells were washed in PBS, fixed with 4% formaldehyde for 30 min and permeabilized with 0.1% saponin (PGN—saponin). After, cells were stained with 50 µg mL^−1^ fluorescent phalloidin conjugate (phalloidin-Atto 565) and To-Pro solution for 50 min at 25 °C in a humidified chamber according to the manufacturer’s instructions. The cell morphology was analyzed in a confocal microscope (Zeiss LSM510 Meta) and captured images were processed using the software Adobe Photoshop 5.5 (Adobe Systems, Inc., Mountain View, CA, USA). Alternatively, the cell area and the nucleus area were manually measured using the ImageJ software (National Institutes of Health, USA) and presented in a bar graph.

### Measurement of reactive oxygen species

ROS levels were measured through CM-H2DCFDA (5-(and-6)-chloromethyl-2′,7′dichlorodihydrofluorescein diacetate acetyl ester) fluorescent dye. A549 and BEAS-2B cells were seeded at a density of 3 × 10^5^ cells/mL in 12-well plates and incubated at 37 °C in a 5% CO_2_ for 12 h. Then, cells were treated or not (control) with 3.8 and 19.0 μM of hmxbato for 4 h. Alternatively, A549 cells were treated for 4 h with 3.8 µM hmxbato and 5 mM NAC (N-acetylcysteine), an thiol-containing antioxidant capable of stimulating glutathione synthesis, thus inhibiting the ROS production^69^. After the treatments, cells were washed twice with PBS and incubated with 10 μM CM-H_2_DCFDA (Invitrogen) and 10 µg/mL propidium iodide for 30 min at 37 ± 0.5 °C in the dark. The cells were analyzed by flow cytometer (CytoFLEX Platform—Beckman Coulter) and more than 10,000 events were acquired. Cells treated with hydrogen peroxide (0.1 mM) for 20 min were used as an internal positive control. The Kaluza software was used for the analysis of results and the graphs represent the average values. This assay was performed in triplicate and three independent experiments were conducted.

### Evaluation of mitochondrial damage (∆Ψ_m_)

Mitochondrial membrane potential (∆Ψ_*m*_) was measured through a specific fluorescent dye that accumulates within active mitochondria—Rhodamine 123 (Rh 123). A549 and BEAS-2B cells (3 × 10^5^ cells mL^−1^) were placed into a 12-well plate and incubated at 37 °C in a 5% CO_2_ for 12 h. After cell adhesion, cells were cultured in the absence (control) or presence of hmxbato (3.8 and 19.0 μM) for 24 h. Following, cells were washed 2 × with PBS and incubated with Rh 123 (15 µg mL^−1^) for 15 min at 37 °C under light protection. Immediately, cells were analyzed by flow cytometer (CytoFLEX Platform—Beckman Coulter) and more than 10,000 events were acquired. The software Kaluza was used to the analysis of results and the graphs represent the average values. This assay was performed in triplicate and three independent experiments were conducted.

### Comet assay

The comet assay was performed according to the protocol proposed by Tice et al.^70^. Briefly, A549 and BEAS-2B (2 × 10^5^ cells/ well) cells in complete DMEM/F12 medium were cultured in 24-well plates for 12 h and, after stabilization, the cells were stimulated with hmxbato (3.8, 1.9 and 0.95 μM), medium (negative control) or methyl methanesulfonate (MMS) (200 μM; positive control) for 4 h. Cell viability was determined by the Trypan blue exclusion technique, using the Countess Automated Cell Counter (Life Technologies, Carlsbad, USA). Cultures with cell viability greater than 70% were subjected to the comet assay, as follows: 20 μL of cell suspension was transferred to agarose-coated slides, covered with coverslips and cooled at 4 °C for 30 min. Then, coverslips were removed; the slides were immersed in freshly prepared lysis solution (2.5 M NaCl, 100 mM EDTA, 10 mM Tris, pH 10) and incubated overnight at 4 °C. Thereafter, the electrophoresis was conducted in a chamber filled with buffer (300 mM NaOH and 1.0 mM EDTA; pH > 13), under standard conditions (25 V; 300 mA; 1.25 V/cm) for 20 min. The slides were then neutralized (0.4 M Tris, pH 7.5), air-dried, and fixed in absolute ethanol for 5 min. Finally, the slides were stained with a GelRed™ solution in PBS (1:10,000, v/v) for 3 min, and analyzed in a fluorescence microscope (Carl Zeiss, Axiostar Plus, Jena, Germany) using a 515–560 nm excitation filter and a 590 nm filter barrier, at 200 × magnification with the aid of the Comet Assay IV software (Perceptive Instruments, Haverhill, UK). One hundred nucleoids were analyzed per sample and, in total, three cultures were performed per treatment (300 nucleoids). The tail intensity, which means the percentage of DNA in the tail, was used as a parameter of DNA damage.

### Analysis of caspase-9 activity in A549 tumor cells

The activation of caspase-9 was measured using the Caspase-Glo 9 Assay kit (Promega-USA) according to the manufacturer's guidelines. The assay provides a luminogenic substrate for caspase-9 in a buffer system. Thus, the addition of the reagent results in cell lysis, followed by the cleavage of caspase and the release of aminoluciferin, a substrate for the enzyme luciferase. Briefly, A549 cells (3 × 10^4^ cells/well) were cultivated in 96-well plate and incubated at 37 °C in a 5% CO_2_ for 12 h. After adhesion, cells were treated or not (control) with 3.8 μM of hmxbato for 24 h. Then, cells were washed with PBS buffer and the kit reagent added in a 1:1 ratio (PBS: kit reagent). The plate was centrifuged at 10 xg for approximately 2 min and incubated at room temperature protected from light for 45 min. Finally, luminescence was evaluated in a Glomax Discover Promega microplate reader. The GraphPad Prism 6 software was used for the analysis of results and the graphs represent the average values. This assay was performed in triplicate and three independent experiments were conducted.

### Analysis of caspase-3 and PARP-1 expression

A549 cells were cultivated in culture bottles (10^7^ cells/bottle) and kept at 37 °C in a 5% CO_2_ until reaching 80% confluence. Subsequently, the cells were fixed and permeabilized with the CytoFix/CytoPerm kit (BD Pharmingen) and incubated for 24 h with complete DMEM/F12 medium in the absence (control) or presence of treatment (3.8 µM hmxbato). After treatment, the cells were incubated with anti-Caspase-3 antibody (Thermo Fisher Scientific, 700,182, 1: 100) and anti-PARP-1 antibody (Sigma-Aldrich, AV33754, 1: 100) for 1 h at 25° C. After incubation with primary antibodies, the cells were washed with PBS and incubated with a goat anti-rabbit IgG secondary antibody conjugated to FITC (1: 200; 656,111, Invitrogen) for 1 h at 25° C and protected from light. The cells were analyzed in quadruplicates by flow cytometry (BD Accuri C6) and two independent experiments were conducted. For each sample, 10,000 events were collected. The graphs were properly analyzed, and the Caspase 3 and PARP-1 expressions determined by the FlowJo software (Treestar). In order to quantify the expression, the median values ​​of the generated histograms were collected and analyzed in the GraphPad Prism Software version 6.01 software (GraphPad Software Inc., La Jolla, CA), being represented by bar graphs.

### DNA docking studies

The crystal structure of the DNA considered as target was the duplex d(CCGGACCCGG/CCGGGTCCGG)_2_ complexed with [Ru(TAP)2(11,12-CN2-dppz)]^2+^, PDB code: 6g8s. This system was considered to validate the efficiency of the molecular docking methodology to describe the correct binding mode and intermolecular interactions between Hmxbato and DNA. The redock procedures were done using the AutoDockTools 1.5.6 (Scanner) interface along with autogrid4 and autodock4 (AD4). To prepare the ligand Ruthenium(II) complex (Hmxbato) to molecular docking, the AutoDockTools 1.5.6 added Gasteiger charges and converted the crystal structures to the format required for AD4 (Sanner, n.d.). The settings used for the Lamarckian genetic algorithm in Autodock were: 150 individuals in a population; 25,000,000 maximum energy evaluations; 27,000 maximum generations; one individual surviving into next-generation; 150 genetic algorithm docking runs; and a ranked cluster analysis was performed on each docking calculation. The Autodock Binding Energy was used for scoring.

### Statistical analysis

The results show data expressed as mean ± standard deviation of assays developed in triplicate by at least three independent experiments. Firstly, the data were checked for a normal distribution. Statistically significant differences between experimental and control samples were determined by one-way ANOVA and Tukey’s multiple comparison test (GraphPad Prism software version 6.01). The data showed statistical significance when value *p < 0.05* (*). Higher significance data were considered for values *p* < 0.01 (**), *p* < 0.001 (***) and *p* < 0.0001 (****).

## Supplementary information

Supplementary Information.
