# Supplementary material for: Ruthenium (II) complex cis-[RuII(ŋ2-O2CC7H7O2)(dppm)2]PF6-hmxbato induces ROS-mediated apoptosis in lung tumor cells producing selective cytotoxicity
Source: Sci Rep. 2020 Sep 21;10:15410. doi: 10.1038/s41598-020-72420-w (PMC7506019; doi:10.1038/s41598-020-72420-w)
Supplement: Supplementary file 1 — Supplementary Information. [file 41598_2020_72420_MOESM1_ESM.docx]

**Ruthenium (II) complex *cis*-[Ru^II^((ŋ^2^-O_2_CC_7_H_7_O_2_)(dppm)_2_]PF_6_ - hmxbato induces ROS-mediated apoptosis in lung tumor cells producing selective cytotoxicity**

Mônica Soares Costa^a^, Yasmim Garcia Gonçalves^b^, Bruna Cristina Borges^c^, Marcelo José Barbosa Silva^c^, Martin Krähenbühl Amstalden^d^, Tássia Rafaella Costa^a^, Lusânia Maria Greggi Antunes^d^, Renata Santos Rodrigues^a^, Veridiana de Melo Rodrigues^a^, Eduardo de Faria Franca^e^, Mariana Alves Pereira Zoia^f^, Thaise Gonçalves de Araújo^f^, Luiz Ricardo Goulart^f^, Gustavo Von Poelhsitz^b^, Kelly Aparecida Geraldo Yoneyama^a*^

^a^ Laboratório de Bioquímica e Toxinas Animais, Instituto de Biotecnologia, Universidade Federal de Uberlândia, UFU, Uberlândia–MG, Brazil;

^b^Instituto de Química, Universidade Federal de Uberlândia, UFU, Uberlândia–MG, Brazil;

^c^Laboratório de Osteoimunologia e Imunologia dos Tumores, Instituto de Ciências Biomédicas, Universidade Federal de Uberlândia, UFU, Uberlândia–MG, Brazil;

^d^Departamento de Análises Clínicas, Toxicologia e Ciências Alimentares, Faculdade de Ciências Farmacêuticas de Ribeirão Preto, Universidade de São Paulo, Avenida do Café s/no CEP 14040-903, Ribeirão Preto, São Paulo, Brazil;

^e^Laboratório de Cristalografia e Química Computacional, Instituto de Química, Universidade Federal de Uberlândia, UFU, Uberlândia–MG, Brazil.

^f^Laboratório de Nanobiotecnologia, Instituto de Biotecnologia, Universidade Federal de Uberlândia, UFU, Uberlândia–MG, Brazil;

**^*^Corresponding author:** Prof. Dra. Kelly Aparecida Geraldo Yoneyama^1^ and Mônica Soares Costa^2^

^1^ Phone: +55 34 3225 8436 #22, Fax: +55 34 3225 8435 – Laboratory address: Pará avenue, 1720 CEP: 38400-902 – Uberlândia, MG, Brazil. E-mail: kelly.tudini@ufu.br

^2^ Phone: +55 34 3225 8436 #22, Fax: +55 34 3225 8435 – Laboratory address: Pará avenue, 1720 CEP: 38400-902 – Uberlândia, MG, Brazil. E-mail: monicacosta.farma@gmail.com.

***Supplementary Data***

*1. Stability analysis of Hmxbato*

The hmxbato complex stability in DMSO was monitored by ^31^P {^1^H} NMR experiments for a 72 h period using 1.0×10^−3^ mol L^−1^ solution of the complex. As seen in Fig. S1 no new signal was detected at the period evaluated. Only two triplet signals at -13.27 and 9.14 ppm were observed. Each triplet signal was attributed to the two chemically and magnetically equal phosphorus present in the complex structure. Based on these results it can be concluded that there was no labilization of any bond of the complex during the analysis time.

**Fig S1.** ^31^P{^1^H} NMR spectra of the Hmxbato complex at 0, 6, 12, 24, 48 and 72 h, in 1.0 mmol L^−1^ in DMSO solution.

*2.*  *Dose-response curve of A549 and BEAS-2B cells*

**Fig S2. Dose-response curve of A549 cells treated with hmxbato complex.** A549 cells were incubated with different concentrations of hmxbato for 24 h. Alternatively, the cells were incubated in presence of DMSO concentrations corresponding to those used as a vehicle in the different concentrations of hmxbato tested. The cytotoxicity was determined by MTT assay. The logs of concentrations showed in graph (x-axis) were converted to molarity and shown in the table below.

| **Concentration (µM)** | **Concentration (M)** | **Concentration log (M)** |
| --- | --- | --- |
| 200 | 0,0002 | -3,698970004 |
| 100 | 0,0001 | -4 |
| 50 | 0,00005 | -4,301029996 |
| 25 | 0,000025 | -4,602059991 |
| 12,5 | 0,0000125 | -4,903089987 |
| 6,25 | 0,00000625 | -5,204119983 |
| 3,125 | 0,000003125 | -5,505149978 |
| 1,5625 | 1,5625E-06 | -5,806179974 |
| 0,78125 | 7,8125E-07 | -6,10720997 |
| 0,390625 | 3,90625E-07 | -6,408239965 |
| 0,1953125 | 1,95313E-07 | -6,709269961 |
| 0,09765625 | 9,76563E-08 | -7,010299957 |

**Fig S3. Dose-response curve of BEAS-2B cells treated with with hmxbato complex.** BEAS-2B cells were incubated with different concentrations of hmxbato for 24 h. Alternatively, the cells were incubated in presence of DMSO concentrations corresponding to those used as a vehicle in the different concentrations of hmxbato tested. The cytotoxicity was determined by MTT assay. The logs of concentrations showed in graph (x-axis) were converted to molarity and shown in the table above.
